# Control of maternal Zika virus infection during pregnancy is associated with lower antibody titers in a macaque model

**DOI:** 10.3389/fimmu.2023.1267638

**Published:** 2023-09-22

**Authors:** Nicholas P. Krabbe, Elaina Razo, Hunter J. Abraham, Rachel V. Spanton, Yujia Shi, Saswati Bhattacharya, Ellie K. Bohm, Julia C. Pritchard, Andrea M. Weiler, Ann M. Mitzey, Jens C. Eickhoff, Eric Sullivan, John C. Tan, Matthew T. Aliota, Thomas C. Friedrich, David H. O’Connor, Thaddeus G. Golos, Emma L. Mohr

**Affiliations:** ^1^ Department of Pediatrics, School of Medicine and Public Health, University of Wisconsin-Madison, Madison, WI, United States; ^2^ Department of Veterinary and Biomedical Sciences, College of Veterinary Medicine, University of Minnesota-Twin Cities, St. Paul, MN, United States; ^3^ Wisconsin National Primate Research Center, University of Wisconsin-Madison, Madison, WI, United States; ^4^ Department of Comparative Biosciences, School of Veterinary Medicine, University of Wisconsin-Madison, Madison, WI, United States; ^5^ Department of Biostatistics and Medical Informatics, School of Medicine and Public Healthy, University of Wisconsin-Madison, Madison, WI, United States; ^6^ Nimble Therapeutics, Inc, Madison, WI, United States; ^7^ Department of Pathobiological Sciences, School of Veterinary Medicine, University of Wisconsin-Madison, Madison, WI, United States; ^8^ Department of Pathology and Laboratory Medicine, School of Medicine and Public Health, University of Wisconsin-Madison, Madison, WI, United States; ^9^ Department of Obstetrics and Gynecology, School of Medicine and Public Health, University of Wisconsin-Madison, Madison, WI, United States

**Keywords:** Zika virus, ZIKV, macaque model, pregnancy, maternal ZIKV infection, congenital Zika syndrome (CZS), maternal antibody response

## Abstract

**Introduction:**

Zika virus (ZIKV) infection during pregnancy results in a spectrum of birth defects and neurodevelopmental deficits in prenatally exposed infants, with no clear understanding of why some pregnancies are more severely affected. Differential control of maternal ZIKV infection may explain the spectrum of adverse outcomes.

**Methods:**

Here, we investigated whether the magnitude and breadth of the maternal ZIKV-specific antibody response is associated with better virologic control using a rhesus macaque model of prenatal ZIKV infection. We inoculated 18 dams with an Asian-lineage ZIKV isolate (PRVABC59) at 30-45 gestational days. Plasma vRNA and infectious virus kinetics were determined over the course of pregnancy, as well as vRNA burden in the maternal-fetal interface (MFI) at delivery. Binding and neutralizing antibody assays were performed to determine the magnitude of the ZIKV-specific IgM and IgG antibody responses throughout pregnancy, along with peptide microarray assays to define the breadth of linear ZIKV epitopes recognized.

**Results:**

Dams with better virologic control (n= 9) cleared detectable infectious virus and vRNA from the plasma by 7 days post-infection (DPI) and had a lower vRNA burden in the MFI at delivery. In comparison, dams with worse virologic control (n= 9) still cleared detectable infectious virus from the plasma by 7 DPI but had vRNA that persisted longer, and had higher vRNA burden in the MFI at delivery. The magnitudes of the ZIKV-specific antibody responses were significantly lower in the dams with better virologic control, suggesting that higher antibody titers are not associated with better control of ZIKV infection. Additionally, the breadth of the ZIKV linear epitopes recognized did not differ between the dams with better and worse control of ZIKV infection.

**Discussion:**

Thus, the magnitude and breadth of the maternal antibody responses do not seem to impact maternal virologic control. This may be because control of maternal infection is determined in the first 7 DPI, when detectable infectious virus is present and before robust antibody responses are generated. However, the presence of higher ZIKV-specific antibody titers in dams with worse virologic control suggests that these could be used as a biomarker of poor maternal control of infection and should be explored further.

## 1 Introduction

Maternal Zika virus (ZIKV) infection during pregnancy results in a wide spectrum of disease in prenatally exposed infants, yet the factors that contribute to differing disease severity are not fully understood. In 2015, a large ZIKV outbreak in the Americas was linked to vertical transmission that resulted in severe birth defects and fetal loss, termed Congenital Zika Syndrome (CZS) (1). CZS occurs in 5-14% of maternal ZIKV infection cases and includes ocular abnormalities, brain anomalies, microcephaly, cranial dysmorphologies, congenital contractures, and hearing loss (2–4). A larger percentage of ZIKV-exposed infants are born without symptoms or may develop neurodevelopmental deficits that manifest in the early years of life, including language, cognitive, and fine motor delays (5–9). The wide range of outcomes observed in prenatally exposed infants highlights the need to better understand the characteristics of maternal ZIKV infection that are associated with differential disease severity.

Some features of maternal ZIKV infection that are associated with worse infant outcomes have already been defined. Asian lineage ZIKV was shown to be the etiological agent of CZS in the Americas (10). Specifically, maternal infection with Asian lineage ZIKV during the first trimester of pregnancy has been identified as a risk factor for more severe infant outcomes compared with infections later in pregnancy (4, 11–13). Different levels of maternal virologic control following Asian lineage ZIKV infection may also explain the differing severity of fetal outcomes. For example, severe fetal pathology was observed concomitant with isolation of replication competent virus from the maternal-fetal interface (MFI) and fetal tissues (14, 15). This may be due to poor maternal virologic control allowing ZIKV to disseminate to this immune-privileged site. Also, prolonged maternal plasma viral RNA (vRNA) burden is associated with more severe fetal outcomes when compared to maternal infections lacking this feature (15–19). Different strains of ZIKV may also be associated with different infant outcomes: infection with low-passage African lineage ZIKV isolates result in more severe fetal pathology compared to infection with isolates of the Asian ZIKV lineage in murine models (20, 21). Pregnancy and infant outcomes of African lineage ZIKV infection have not been well defined in humans yet, though non-human primate (NHP) models have found that infection with a high dose of African lineage ZIKV during the first trimester results in high rates of fetal demise while low dose infection does not (22–24). These findings suggest that several factors of maternal ZIKV infection could contribute to the varying levels of disease severity seen in prenatally exposed infants, including the timing of infection during pregnancy, the overall level of maternal virologic control following infection, and the lineage of ZIKV that is responsible for infection.

Differences in the maternal immune responses following infection may explain some of the variability in maternal control of ZIKV infection. Following the ZIKV outbreak in the Americas, a great deal of attention was paid to understanding correlates of protection that could be used to develop a vaccine to prevent congenital ZIKV infection (25, 26). Several ZIKV vaccine candidates were proposed that largely elicited antibody responses that proved to be protective against ZIKV infection during pregnancy in murine and NHP models (27–31). Additionally, several groups have identified potential monoclonal antibodies (mAb) that, when administered prior to infection, target specific ZIKV epitopes and are protective against congenital infection (32–35). Despite this effort, there are no licensed vaccines or prophylactic mAb therapies that are available for use in pregnant people. In contrast, an area of research that remains largely unexplored is understanding the natural maternal antibody response following ZIKV infection, and how this response associates with maternal virologic outcomes.

Rhesus macaques are a good translational model of human ZIKV infection because they are able to recapitulate maternal infection outcomes, and they develop similar neutralizing antibody responses to ZIKV infection (36). They also avoid utilizing human cohorts that are often in regions where different flaviviruses co-circulate. All flaviviruses exhibit a high degree of antigenic similarity, and infection with one flavivirus is known to generate antibodies that cross react with epitopes on heterotypic viruses, confounding serological assays (37, 38). This makes it challenging to accurately determine the natural ZIKV-specific immune response following ZIKV infection in human cohort studies. Previous flavivirus exposure could also modulate maternal ZIKV infection control making it difficult to accurately draw conclusions about the role of the maternal ZIKV-specific antibody response in controlling maternal infection (39, 40). Additionally, human studies may not capture the full spectrum of ZIKV-specific antibody responses seen in both severe infection and mild or asymptomatic infection, because women with no symptoms may not present for medical care and would not be enrolled in studies (37). The inability to accurately determine the timing of maternal infection in human studies also makes it challenging to track the maternal antibody responses over the full course of infection. To avoid these inherent limitations associated with human cohort studies, we opted to utilize a well-characterized translational macaque model of ZIKV infection during pregnancy (41, 42).

Here, we describe the virologic outcomes for 18 pregnant dams infected in the first trimester with an Asian-lineage ZIKV isolate (PRVABC59). We report plasma vRNA and infectious virus kinetics, as well as vRNA burden in the MFI tissues at delivery for each dam. We also compare the magnitude of the ZIKV-specific antibody responses and the breadth of linear epitopes recognized in the ZIKV polyprotein between the two virologic control groups to determine if there were specific antibody responses associated with virologic control.

## 2 Materials and methods

### 2.1 Study design

18 pregnant Indian-origin rhesus macaques (*Macaca mulatta*) were inoculated subcutaneously over the cranial dorsum with 1x10^4^ plaque forming units (PFU) of Zika virus/*H.sapiens-tc/PUR/2015/PRVABC59_v3c2* (PRVABC59, GenBank: KU501215) during the first trimester (term is 165 ± 10 days). First trimester inoculations were chosen to model the more severe outcomes of congenital ZIKV infection seen in human cases (4, 11–13). All dams were flavivirus naïve and free of *Macacine herpesvirus 1* (Herpes B), simian retrovirus type D (SRV), simian T-lymphotropic virus type 1 (STLV), and simian immunodeficiency virus (SIV). The preparation of the ZIKV-PRVABC59 stock was described previously (43). Dams were inoculated with ZIKV in the first trimester (at ~30 gestational days (gd), n= 9, or at ~45gd, n= 9) and blood was drawn for ZIKV qRT-PCR daily for 10 days following inoculation, then twice weekly until viremia cleared, then weekly until the end of pregnancy, except for dam 044-117 due to COVID-19 restrictions (**Supplementary Figures 1A, B**). Dams underwent Cesarean section (C-section) at approximately 160gd, about 6 days earlier than the average gestational age of a natural birth at the Wisconsin National Primate Research Center (WNPRC) to ensure that maternal-fetal interface tissues could be collected for virologic studies. Two dams delivered by natural delivery (044-109 and 044-131) just prior to their scheduled C-sections and their maternal-fetal interface tissues were unable to be collected. Four of these ZIKV pregnancies have been described earlier (39).

### 2.2 Ethics statement

All monkeys are cared for by the staff at the WNPRC in accordance with the regulations and guidelines outlined in the Animal Welfare Act and the Guide for the Care and Use of Laboratory Animals, the recommendations of the Weatherall report (44), and the principles described in the National Research Council’s Guide for the Care and Use of Laboratory Animals (45). The University of Wisconsin - Madison Institutional Biosafety Committee approved this work under protocol number B00000764.

### 2.3 Care and use of macaques

All animals were housed in enclosures with required floor space and fed using a nutritional plan based on recommendations published by the National Research Council (45). Dams were fed a fixed formula, extruded dry diet with adequate carbohydrate, energy, fat, fiber, mineral, protein, and vitamin content. Macaque dry diets were supplemented with fruits, vegetables, and other edible objects (e.g., nuts, cereals, seed mixtures, yogurt, peanut butter, popcorn, marshmallows, etc.) to provide variety to the diet and to reinforce species-specific behaviors such as foraging. To further promote psychological well-being, animals were provided with food enrichment, structural enrichment, and/or manipulanda. Environmental enrichment objects were selected to minimize chances of pathogen transmission from one animal to another and from animals to care staff. While on study, all animals were evaluated by trained animal care staff at least twice each day for signs of pain, distress, and illness by observing appetite, stool quality, activity level, and physical condition. Animals exhibiting abnormal presentation for any of these clinical parameters were provided appropriate care by attending veterinarians. Prior to all minor/brief experimental procedures, macaques were sedated using ketamine anesthesia and monitored regularly until fully recovered from anesthesia.

The female macaques were co-housed with a compatible male and observed daily for menses and breeding. Pregnancy was detected by ultrasound examination of the uterus at approximately 20-24gd following the predicted day of ovulation. The gd was estimated (+/- 2 days) based on the dam’s menstrual cycle, observation of copulation, and the greatest length of the fetus at initial ultrasound examination which was compared to normative growth data in this species (46). For physical examinations, virus inoculations, ultrasound examinations, blood or swab collections, the dam was anesthetized with an intramuscular dose of ketamine (10 mg/kg). Blood samples from the femoral or saphenous vein were obtained using a vacutainer system or needle and syringe. Pregnant macaques were monitored daily prior to and after viral inoculation for any clinical signs of infection (e.g., diarrhea, inappetence, inactivity, fever and/or atypical behaviors).

### 2.4 Plasma and serum preparation

For plasma, whole blood was collected from the dams in EDTA-treated vacutainer tubes. The whole blood was then separated using Ficoll density centrifugation for 30 minutes at 1,860xg to isolate peripheral blood mononuclear cells (PBMCs) and plasma, or it was centrifuged at 1,400xg for 15 minutes to isolate plasma. The plasma layer was removed and centrifuged at 670xg for 8 minutes to remove any remaining cell debris. For serum, whole blood was collected in serum separator tubes (SST). The tube was spun at 1,400xg for 20 minutes and the serum layer was removed and centrifuged at 670xg for 8 minutes to remove any remaining cell debris. Processed plasma and serum samples were aliquoted and frozen at -80 °C.

### 2.5 Viral RNA (vRNA) isolation from blood and tissues and RT-qPCR

RNA was extracted from 300 µL of plasma using the Viral Total Nucleic Acid Purification kit (Promega, Madison, WI, USA) on a Maxwell 48 RSC instrument. RT-qPCR was performed as previously described (42). Biopsies from maternal-fetal interface tissues (placenta, decidua, and chorionic plate) were preserved with RNAlater (Invitrogen, Carlsbad, CA, USA) at 4 °C for 24–72 hours before the RNAlater was removed and the tissue biopsies were frozen at −80 °C. RNA was isolated from maternal-fetal interface tissue biopsies using a method described by Hansen et al. (47) and previously described in detail (48). The tissue vRNA load was calculated as the mean of duplicates, or the mean of multiple replicates if the sample was run more than once. The RT-qPCR limits of detection are 150 copies/mL from plasma and estimated to be 3 copies/mg from tissue. The percent of vRNA-positive placental, decidual, and chorionic plate biopsies was calculated by dividing the number of vRNA-positive biopsies from each tissue by the total number of biopsies from each tissue that were assayed.

### 2.6 Viral Quantification by plaque assay

Titrations for replication competent virus quantification from blood specimens was completed via plaque assays on Vero cells (ATCC CCL-81). Serum or plasma samples (0.15mL) were serially diluted 10-fold from 1:10-10,000 in 1X DMEM (Gibco)-2% fetal bovine serum (FBS) (v/v) and added to Vero cells in duplicate in 12-well culture plates. The plates were incubated for 1 hour at 37 °C 5% CO_2_ to allow for effective virus adsorption, gently rocking every 15 minutes to ensure an even distribution of sample across the cell monolayer. Following incubation, the monolayers were overlaid with 1mL of overlay medium containing a mixture of 1.2% microcrystalline cellulose (Beantown Chemical, Hudson, NH, USA) and 2X DMEM (Gibco) with 10% FBS (v/v), 2% GlutaMAX (Gibco), and 2% penicillin/streptomycin. Cells were incubated at 37 °C in 5% CO_2_ for 72 hours to allow for plaque formation. After 72 hours, the overlay medium was discarded, and the cells were fixed with ice-cold methanol for 20 minutes at 4 °C. The monolayers were then stained with 0.5% crystal violet in diH_2_O with 20% methanol (v/v) for 5 minutes at room temperature and the number of plaques was counted.

### 2.7 Plaque reduction neutralization tests (PRNTs)

Macaque serum samples were screened for ZIKV-specific neutralizing antibodies (nAbs) using a ZIKV PRNT at three timepoints: pre-infection, 27-38 days post-infection (DPI), and 98-136 DPI (**Supplementary Table 1**). Endpoint titrations of reactive sera were performed against ZIKV (PRVABC59) as previously described (49). Briefly, ZIKV was mixed with 2-fold serial dilutions of sera for 1 hour at 37 °C prior to being added to Vero cells. Neutralization curves were generated using R statistical language (R Core Team 2022), version 4.2.2. The resulting data were analyzed using a 4-parameter dose-response model by the R extension package ‘drc’ (50) to estimate the dilution of serum required to inhibit 90% of infection (PRNT_90_) and 50% of infection (PRNT_50_). Independent samples with PRNT_90_ values lower than the lowest serum dilution were classified as below the limit of detection for the assay.

### 2.8 ZIKV whole-virion binding IgG antibody assay

Macaque serum samples were screened for ZIKV-specific IgG binding antibodies over the course of pregnancy at numerous timepoints (**Supplementary Table 1**), similar to a previously defined protocol (51). High-binding 96-well ELISA plates (Greiner; Monroe, NC) were coated with 50 ng/well of recombinant anti-flavivirus 4G2 antibody (clone D1-42-4-15, Absolute Antibody; Wilton Centre, UK) (**Supplementary Table 2**) diluted in 0.1M sodium carbonate buffer (pH 9.6) and incubated overnight at 4 °C. Plates were blocked with 1X tris-buffered saline (TBS) containing 0.05% Tween-20 and 5% normal goat serum (Invitrogen, Waltham, MA) for 1 hour at 37 °C, followed by incubation with 5.5 x 10^4^ plaque forming units (PFU)/well of ZIKV (PRVABC59) for 1 hour at 37 °C. Plates were washed 3 times with 1X TBS containing 0.2% Tween-20. Macaque serum samples were added to the wells of the plate in duplicate using a 4-fold dilution series ranging from 1:12.5 to 1:204,800 and incubated for 1 hour at 37 °C. Plates were washed 3 times with 1X TBS containing 0.2% Tween-20. Horseradish peroxidase (HRP)-conjugated mouse anti-monkey IgG antibody (Southern BioTech; Birmingham, AL) (**Supplementary Table 2**) was added to the wells at a 1:4,000 dilution and incubated at 37 °C for 1 hour. Plates were washed 3 times with 1X TBS containing 0.2% Tween-20, followed by the addition of SureBlue reserve TMB substrate (KPL; Gaithersburg, MD). Reactions were terminated by stop solution (KPL; Gaithersburg, MD) after a 10-minute incubation per plate in the dark. Optical density was detected at 450nm (OD450) on a SpectraMax M5e plate reader (Molecular Devices; San Jose, CA). IgG binding antibody curves were generated using R statistical language (R Core Team 2022), version 4.2.2. Dose-response models (4-parameter) were generated and the dilution of serum required to achieve a 90% and 50% reduction in maximum OD450 reading (EC_90_ and EC_50_, respectively) were estimated using the R extension package ‘drc’ (50). Samples were classified as below the limit of detection if they had EC_90_ values lower than the lowest serum dilution.

### 2.9 ZIKV IgM ELISA

A commercially available ZIKV IgM ELISA kit (EI 2668-9601 M; Euroimmun, Lübeck, Germany) was performed on serum samples from several time points (**Supplementary Table 1**). If samples remained positive for ZIKV IgM at the 27-35 DPI time point, then a 50-60 DPI sample was run and if samples remained positive, an 80-91 DPI sample was run. The protocol was performed as specified by the manufacturer. Briefly, all the samples were diluted 1:100 in sample dilution buffer containing RF/IgG absorbent, as recommended by the manufacturer. Immediately following addition of the stop solution, the OD of each sample was determined at 450 nm. The OD readings of the samples were divided by the OD reading of the calibration sample to determine a sample ratio. Samples with a ratio greater than 1.1 were considered positive for ZIKV IgM and samples with a ratio less than 0.8 were considered negative for ZIKV IgM. Any sample with a ratio between 0.8 and 1.1 was considered borderline positive.

### 2.10 Peptide microarray assays

#### 2.10.1 Peptide array design and synthesis

The viral polyprotein sequence for ZIKV (PRVABC59) (GenBank accession AMC13911) was submitted to Nimble Therapeutics (Madison, WI), for development into a peptide microarray. Proteins were tiled as non-redundant 16 amino acid peptides overlapping by 15 amino acids. As described previously, peptide sequences were synthesized *in situ* with a Roche Sequencing Solutions Maskless Array Synthesizer (MAS) by light-directed solid-phase peptide synthesis using an amino-functionalized support (Greiner Bio-One) coupled with a 6-aminohexanoic acid linker and amino acid derivatives carrying a photosensitive 2-(2-nitrophenyl) propyloxycarbonyl (NPPOC) protection group (Orgentis Chemicals) (52). Unique peptides were synthesized in random positions on the array to minimize impact of positional bias. Each array is comprised of twelve subarrays, where each subarray can process one sample and each subarray contains up to 392,318 unique peptide sequences.

#### 2.10.2 Peptide array sample binding

Macaque serum samples from a subset of pregnant dams (n=11) were screened for both IgM and IgG antibody binding to the peptide array at specific post-infection timepoints: 13-17 and 21-24 DPI for IgM and 27-31 and 108-135 DPI for IgG (**Supplementary Table 1**). This subset was selected based on the dams that had completed pregnancy at the time the peptide array was performed. Samples were diluted 1:50 in binding buffer (0.01M Tris-Cl, pH 7.4, 1% alkali-soluble casein, 0.05% Tween-20). Diluted serum aliquots and negative controls (binding buffer only) were bound to arrays overnight for 16–20 hours at 4 °C. After binding, the arrays were washed 3x in wash buffer (1x TBS, 0.05% Tween-20), 10 minutes per wash. Primary sample binding was detected via a fluorescently labeled anti-primate IgM or IgG antibody (**Supplementary Table 2**). The secondary antibody was diluted in secondary binding buffer (1x TBS, 1% alkali-soluble casein, 0.05% Tween20) and incubated with arrays for 3 hours at room temperature, then washed 3x in wash buffer (10 minutes per wash) and 30 seconds in reagent-grade water. The IgM antibody was diluted 1:20,000 and the IgG antibody was diluted 1:40,000. Fluorescent signal of the secondary antibody was detected by scanning at 635 nm at 2 µm resolution and 4% gain, using an InnoScan 1100 AL microarray scanner (Innopsys).

#### 2.10.3 Peptide array analysis and optimization

Peptide array data were processed and analyzed using methods previously described (53) and are described briefly here, with modifications for our dataset.

##### 2.10.3.1 Data pre-processing

Fluorescent intensities were log2 transformed for analyses.

##### 2.10.3.2 Data normalization

Normalization was performed to reduce non-biological sources of bias and improve the comparability of signal intensities across slides. We normalized based on physicochemical properties of each peptide (i.e. lipophilicity, polarizability, polarity, electronegativity, electrophilicity), which are represented by a unique Z-scale score for each amino acid, as previously described by (53). We built a Z-scale peptide matrix based on all peptide sequences in the array. The five Z-scale values (ε*_pj_*, *j* = 1,…,5) for each specific peptide (*p*) were calculated by summing the five Z-scale values for the individual amino acids that make up the peptide, so that each peptide has a unique pattern of five Z-scale values. Given the peptide Z-scale values, the background fluorescent intensities for each peptide (*y_p_*) were modeled as:


$$y_{p}=\;\beta_{p}+\;\sum_{j=1}^{5}\beta_{j}\varepsilon_{jp}+\;\in_{p}$$

$\in_{p}$ is a scale student-t distribution with 4 degrees of freedom, $\beta_{p}$ is an intercept term, and $\beta_{j}$ is the overall effect of the *j*-th physiochemical property. The background fluorescence (*y_p_*) from the blank (no serum sample on the array) is subtracted from the raw fluorescent intensity (*y*) for all of the 3,404 different peptides from the ZIKV (PRVABC59) polyprotein sequence utilized in this array to obtain normalized fluorescence intensity. The normalized fluorescent intensities are used for subsequent analyses.

##### 2.10.3.3 Definition of positive peptides

A thresholding method was applied to identify true positive peptides and reduce the identification of false positives, as previously described (53). Briefly, we assumed that the distributions of fluorescent intensities are symmetric about zero with a positive skew when true binding is present. Using the area of the left tail as an estimate of the false positive rate, we estimated the false discovery rate as the area of the left tail divided by the area of the right tail, since the right tail includes false positives and true positives. To identify a fluorescence threshold (*T*) that determines the area of the tails, we iterated through values of *T* to calculate the false discovery rate for each sample (*F_s_(T)*) by the following equation with given threshold *T*:


$$F_{s}\left(T\right)=\left(p\;:\;y_{sp}^{d}<\;-T\right)/(p\;:\;y_{sp}^{d}>T)$$

The final false discovery rate for a given threshold *T*, denoted *F(T)*, is the median *F_s_(T)* for all samples, s. The final threshold *T* was selected as the *T* minimizing the difference, |*F(T) – f|*, where *f* is the target false discovery rate of 0.05. This process was repeated for IgG and IgM samples separately, providing a threshold for IgG (3.795) and a threshold for IgM samples (1.585). The peptides with fluorescent intensities *y_sp_(d)* greater than the fluorescence threshold *T* are identified as positive peptides.

##### 2.10.3.4 Definition of linear epitopes

We defined a continuous linear epitope as a positive peptide adjacent to one or more other positive peptides. A single positive peptide with no adjacent positive peptides does not constitute an epitope with this definition. Unique epitopes are defined as epitopes >1 space away from another epitope. The total count of positive epitopes in the total ZIKV polyprotein and each individual ZIKV viral protein (with viral protein bounds defined in **Supplementary Table 3**) for each dam at each timepoint was calculated and termed the “epitope count”.

##### 2.10.3.5 Epitope mapping approach

To determine the prevalence of specific epitopes recognized by the antibody responses of the dams, we calculated the percentage of dams with reactive peptides, defined as peptides with fluorescent intensity values above the threshold, within all of the identified linear epitopes. We first mapped all linear epitopes to their location in the ZIKV polyprotein. We then determined the percentage of dams that shared reactivity to each peptide within those epitopes. Epitope maps were generated for each ZIKV protein (**Supplementary Table 3**). Additionally, envelope and NS1 were broken down into their previously described sub-domains to further characterize the antibody response to these proteins (54, 55).

### 2.11 Statistical analysis

Figure generation and all statistical analyses were performed using R statistical language (R Core Team 2022), version 4.2.2, or SAS software (SAS Institute, Cary NC), version 9.4. Plasma viral load curves were generated for each dam. From these curves, the area under the curve (AUC), timing of peak vRNA load, magnitude of peak vRNA load, and duration of plasma vRNA burden were determined for each dam. The AUCs were calculated using the trapezoid rule. The percentage of vRNA-positive biopsies from each MFI tissue was determined by dividing the number of vRNA-positive biopsies from the total number of biopsies from that tissue submitted for qRT-PCR. A latent class analysis (LCA) clustering algorithm was performed using area under the curve values, durations of plasma vRNA burden, and the percentage of total MFI biopsies that were vRNA-positive to determine if individual dams clustered based on maternal virologic parameters. The number of classes were determined by sequentially using a likelihood ratio test. The resulting clusters were used in all downstream statistical analyses. All statistical comparisons between the clusters were performed using robust nonparametric Mann-Whitney tests. Statistical comparisons between IgM and IgG linear epitope counts across the different ZIKV proteins (when all dams were treated as a single population) were performed using a nonparametric Kruskal-Wallis test followed by a *post-hoc* pairwise Mann-Whitney test. All reported P-values are two-sided and P<0.05 was used to define statistical significance.

## 3 Results

### 3.1 Maternal ZIKV infection results in dams with differing levels of virologic control

**Figure 1:**
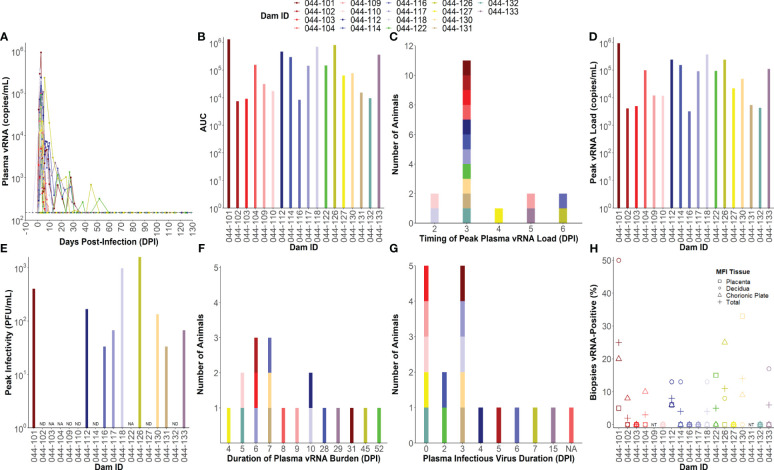
Individual maternal virologic outcomes. **(A)** Maternal plasma viral RNA (vRNA) loads throughout pregnancy determined by RT-qPCR. The lower limit of detection (LLoD) for the assay (150 copies/mL) is represented by a dotted line. All vRNA loads below the LLoD are set equal to the LLoD. **(B)** Area under the curve (AUC) values calculated from maternal plasma vRNA loads throughout pregnancy. **(C)** Timing of peak plasma vRNA loads is denoted by a histogram. **(D)** Peak plasma vRNA loads as determined by RT-qPCR. **(E)** Infectious virus titers determined via plaque assay from blood collected on the same day as peak vRNA load. Infectious virus titers are expressed as plaque forming units (PFU)/mL. “NA” = not applicable and indicates that the dam did not have any sample remaining from the day of peak vRNA load to test. “ND” = infectious virus was not detected on the day of peak vRNA load. **(F)** Duration of plasma vRNA burden is denoted as a histogram indicating the last day where plasma ZIKV vRNA levels were above the LLoD. **(G)** Duration of detectable infectious virus is denoted by a histogram indicating the last day where infectious virus was detected in the blood via plaque assay. “NA” = not applicable and indicates that the dam did not have any sample remaining to test. **(H)** The percentage of maternal-fetal interface (MFI) biopsies from the placenta, decidua, chorionic plate, and all three combined (total) that were vRNA-positive at delivery. The LLoD for tissue samples is 3 copies/mg of tissue. “NT” = not tested because the animal had a natural birth.

All ZIKV-infected dams (n= 18) developed detectable plasma viral RNA (vRNA) loads between 1 and 4 days post-infection (DPI) with area under the curve (AUC) values that ranged from 10^4^ to 10^6^ (**Figures 1A, B**). Dams had plasma vRNA loads that peaked between 2 and 6 DPI and ranged from 10^3.5^ to 10^6^ copies/ml (**Figures 1C, D**). Infectious virus titers from the day of peak vRNA load were determined via plaque assay for dams with available blood specimens at this time point (n= 15). Infectious virus was detected in the blood of 9 of the 15 dams tested on the day of peak vRNA load with titers ranging from 33 to 1,570 plaque forming units (PFU)/mL (**Figure 1E**). The duration of plasma vRNA burden, defined as the last day of a plasma viral load above the lower limit of detection (150 copies/mL), ranged from 4 DPI to 52 DPI (**Figure 1F**). Half of the dams had plasma vRNA duration ≤7 DPI, and half had plasma vRNA duration of ≥8 DPI. The duration of detectable infectious virus in the blood, defined as the last day of a positive plaque assay, was determined for 17/18 dams by testing samples from the last day of a positive vRNA load and then working backwards to determine the latest time point that infectious virus is detectable (044-104 did not have any remaining blood specimens to perform plaque assays on). The majority of the dams had infectious virus that became undetectable by 7 DPI, five dams never had detectable infectious virus, and one dam (044–133) had infectious virus that remained detectable until 15 DPI (**Figure 1G**). The presence of vRNA in the maternal-fetal interface (MFI) tissues (from placental, decidual, and chorionic plate biopsies) varied between individual dams at the time of delivery, with some dams having no detectable vRNA in the MFI, and another having up to 25% of all MFI biopsies positive for vRNA (**Figure 1H**).

**Figure 2:**
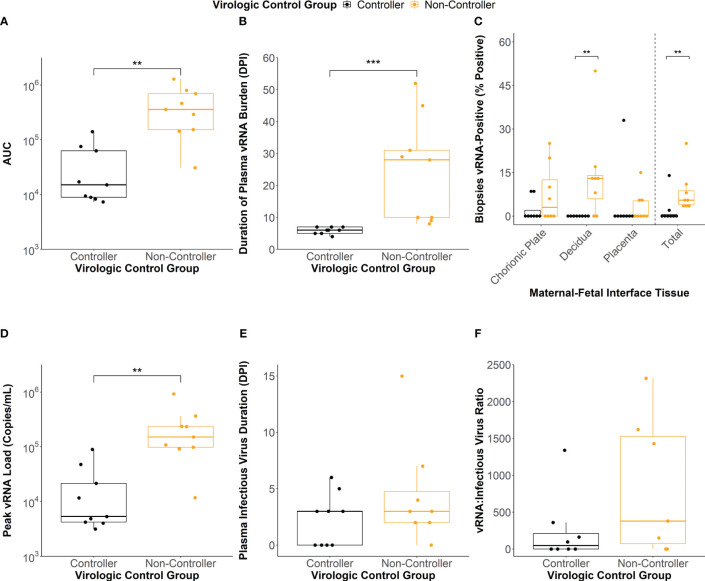
Maternal virologic outcomes based on virologic control status. **(A)** Area under the curve (AUC) values of the viral RNA (vRNA) loads graph. **(B)** Duration of plasma vRNA burden defined as the last day with a plasma vRNA load above the lower limit of detection (150 copies/mL). **(C)** Percentage of vRNA-positive chorionic plate, decidual, placental, and total biopsies from the time of delivery. **(D)** Peak plasma vRNA loads. **(E)** Duration of infectious virus in the plasma determined via plaque assay. Dam 044-104 (non-controller) did not have any remaining sample to assay. **(F)** vRNA to infectious virus ratios were determined for each dam from the day of peak vRNA load. The ratio was determined by dividing the vRNA copies/mL by the infectious virus PFU/mL. Dams with undetectable infectious virus titers were assigned a ratio of 0. Dams 044-103 (controller), 044-104 (non-controller), and 044-122 (non-controller) did not have any sample remaining from the day of peak plasma vRNA load to conduct a plaque assay. Statistically significant differences between virologic control groups were determined using a Mann-Whitney U test (*p<0.05, **p<0.01, ***p<0.001).

To define the association between antibody responses and virologic outcomes of maternal ZIKV infection, we clustered the individual dams using a latent class analysis (LCA) based on the AUC of the viral load kinetics (**Figure 1B**), the duration of plasma vRNA burden (**Figure 1F**), and the percentage of total MFI biopsies that were vRNA-positive at the time of delivery (**Figure 1H**). Two distinct clusters of dams were identified that differed significantly across these three viral parameters (**Figures 2A–C**; **Supplementary Table 4**). The dams comprising the cluster with lower AUCs (**Figure 2A**, p<0.01), shorter durations of plasma vRNA burden (**Figure 2B**, p<0.001), and lower percentages of total MFI biopsies that were vRNA-positive at delivery (**Figure 2C**, p<0.01) are referred to as viral controllers (n= 9), while the other dams are referred to as viral non-controllers (n= 9). Viral controllers also had significantly lower percentages of decidual biopsies that were vRNA-positive at delivery (p<0.01) and lower trending percentages of chorionic plate and placental biopsies that were vRNA-positive at delivery (**Figure 2C**). The two virologic control groups also significantly differed in the magnitude of peak plasma vRNA loads (**Figure 2D**, p<0.01). All viral controllers resolved both vRNA and detectable infectious virus from the blood by 7 DPI, whereas the viral non-controllers cleared detectable infectious virus from the blood by 7 DPI (with the exception of 044-133) but had vRNA that persisted longer (**Figures 2B, E**). Notably, 5/9 (56%) viral non-controllers had prolonged plasma vRNA burdens that lasted for at least 28 DPI (**Figure 2B**). Infectious virus titers in the blood from the day of peak vRNA load were compared with the vRNA copies/mL to determine a vRNA to infectious virus ratio. Eight viral controllers and seven viral non-controllers had sample remaining to assess infectious virus at the time of peak vRNA load. Although not significant, the viral controllers had lower trending vRNA to infectious virus ratios on the day of peak vRNA load compared to the viral non-controllers (**Figure 2F**). This suggests that higher vRNA loads in the viral non-controllers do not necessarily correlate with a proportional increase in infectious virus titers.

### 3.2 Viral controllers have lower ZIKV-specific binding and neutralizing antibody titers

**Figure 3:**
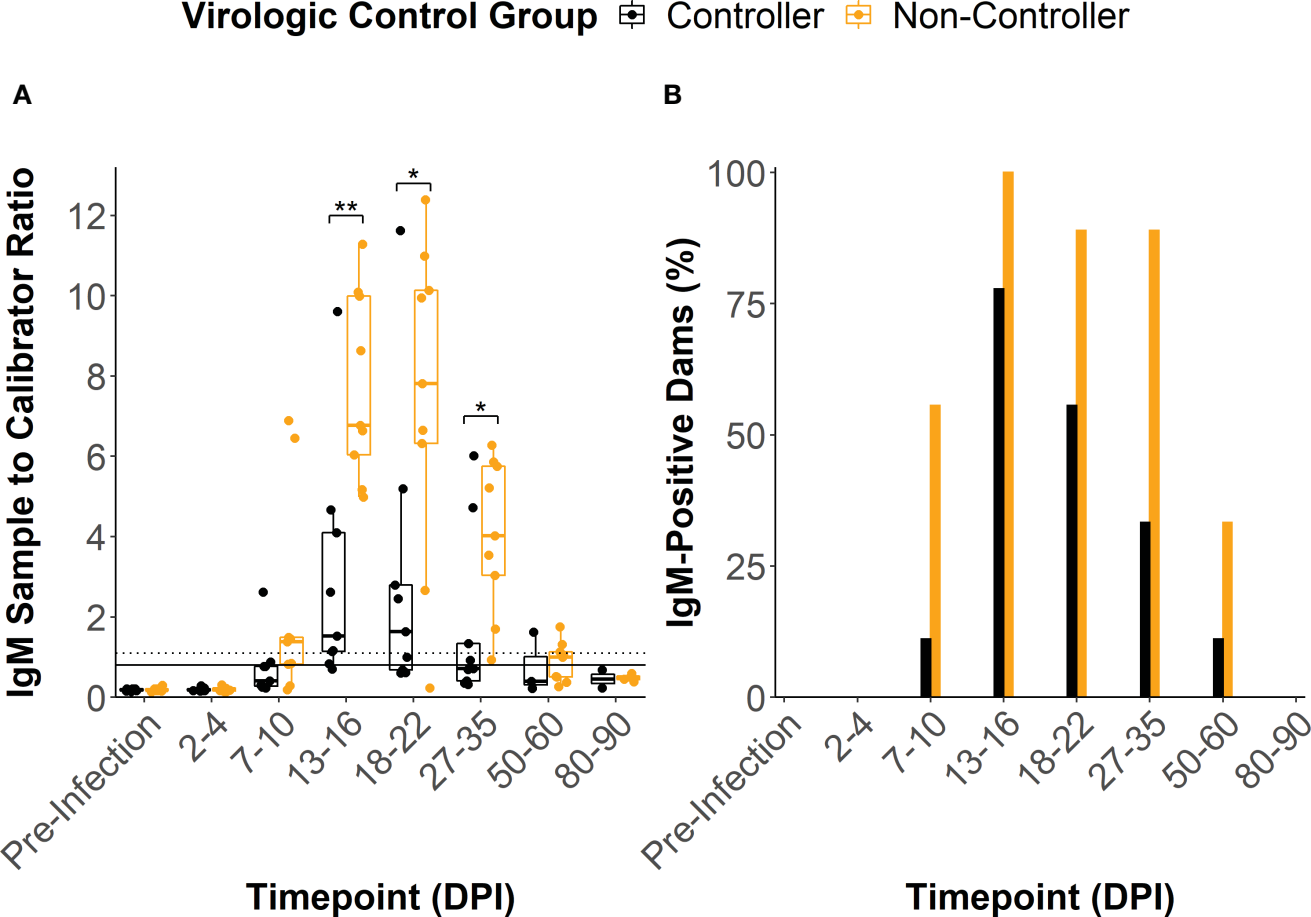
ZIKV-specific IgM antibody dynamics. **(A)** The sample to calibrator ratio for each dam was determined at several time points by dividing the optical density reading at 450 nm (OD450) of the experimental sample by the OD450 of the kits calibrator sample. A ratio that is ≥ 1.1 indicates a positive IgM sample (dotted line), a ratio between 1.1 and 0.8 indicates a sample that is borderline positive, and a ratio that is< 0.8 indicates a negative sample (solid line). Dams were divided based on their virologic control status. **(B)** The percentage of dams in each group that were considered to be positive for ZIKV-specific IgM at each time point was determined by dividing the number of positive animals within the group by the total number of animals within the group. Statistically significant differences between virologic control groups were determined using a Mann-Whitney U test (*p<0.05, **p<0.01, ***p<0.001).

ZIKV-specific binding IgM antibody responses developed by 13-16 DPI in all but two of the infected dams, with peak IgM responses occurring between 13 and 22 DPI (**Figure 3A**). One dam (044–103) never developed a conclusively positive IgM response but was considered borderline positive at 13-16 DPI. The viral controllers had significantly lower IgM response at 13-16 DPI, 18-22 DPI, and 27-35 DPI when compared to the viral non-controllers (**Figure 3A**). Additionally, the viral controllers generally had a lower percentage of IgM positive dams at all timepoints (**Figure 3B**). There was some degree of prolonged IgM detection in both the viral controllers and non-controllers out past 50 DPI (**Figures 3A, B**).

**Figure 4:**
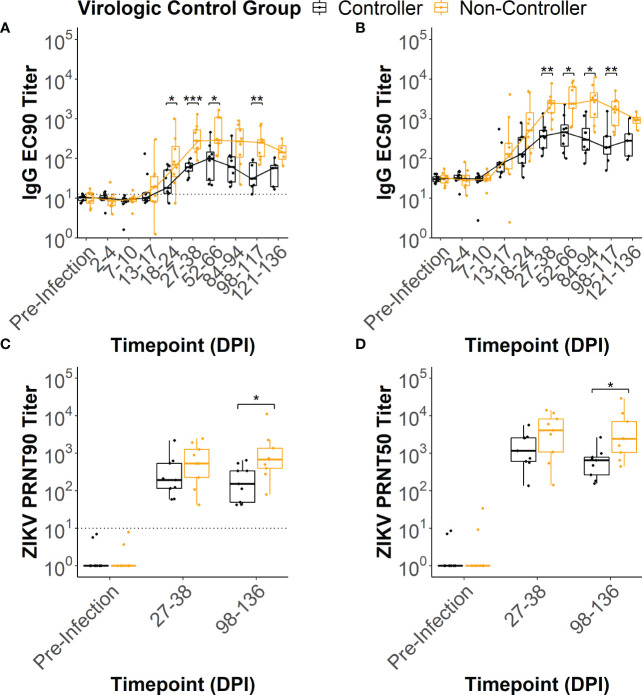
ZIKV-specific IgG binding and neutralizing antibody dynamics. ZIKV-specific IgG binding antibody EC_90_ and EC_50_ titers were estimated from the raw IgG binding antibody curves (**Supplementary Figure 2**) for each animal at multiple timepoints post-infection. Similarly, ZIKV-specific neutralizing antibody PRNT_90_ and PRNT_50_ titers were estimated from the raw neutralizing antibody curves (**Supplementary Figure 4**). The **(A)** EC_90_ and **(B)** EC_50_ titers for the two virologic control groups were compared at each timepoint tested. The limit of detection is denoted by a dotted line on the EC_90_ figure. The **(C)** PRNT_90_ and **(D)** PRNT_50_ titers for the two virologic groups were compared at each timepoint tested. The limit of detection is denoted by a dotted line on the PRNT_90_ figure. Statistically significant differences between the virologic control groups were determined using a Mann-Whitney U test (*p<0.05, **p<0.01, ***p<0.001).

In all of the dams, ZIKV-specific IgG binding antibody (bAb) responses developed by 2-4 weeks post-infection (**Supplementary Figures 2, 3**). IgG bAb dynamics varied across the dams with some developing a robust bAb response that persisted throughout pregnancy and others developing a bAb response that slowly waned over the course of pregnancy (**Supplementary Figures 3A, B**). Dams also varied in the magnitude of peak IgG bAb titers and the time it took to reach those titers. To determine if IgG bAb titer patterns were associated with the level of ZIKV infection control, we compared the effective dilution of serum required to reduce the maximum optical density reading by 90% and 50% (EC_90_ and EC_50_, respectively) between the viral controllers and non-controllers. The viral controllers had significantly lower EC_90_ titers beginning around 18-24 DPI (p<0.05) that continued at 27-38 DPI (p<0.001), 52-66 DPI (p<0.05) and 98-117 DPI (p<0.01) compared to the viral non-controllers (**Figure 4A**). Similarly, EC_50_ titers were significantly lower for the viral controllers at 27-38 DPI (p<0.01), 52-66 DPI (p<0.01), 84-94 DPI (p<0.01), and 98-117 DPI (p<0.01) compared to the non-controllers (**Figure 4B**). These antibody titer patterns indicate that viral controllers began exhibiting significantly lower ZIKV-specific IgG bAb titers around 27-38 DPI compared with viral non-controllers that persisted until just before delivery.

All dams had neutralizing antibody (nAb) titers below the limit of detection at the pre-infection timepoint (**Supplementary Figures 4, 5**). By 27-38 DPI, all dams developed a ZIKV-specific nAb response (**Supplementary Figures 5C, D**) that persisted at 98-136 DPI (**Supplementary Figures 5E, F**). To determine if nAb titers were associated with the level of virologic control, we compared the dilution of serum required to reduce the number of plaques by 90% and 50% (PRNT_90_ and PRNT_50_, respectively) between the viral controllers and viral non-controllers. Both the PRNT_90_ and PRNT_50_ titers were significantly lower for the viral controllers at 98-136 DPI (**Figures 4C, D**) when compared to the viral non-controllers (p<0.05). Despite nAb titers that trended lower for the viral controllers at 27-38 DPI, PRNT_90_ and PRNT_50_ titers did not differ significantly between the two virologic control groups (**Figures 4C, D**).

### 3.3 Breadth of the ZIKV-specific linear epitope response did not differ based on virologic control status

To determine how linear epitope profiles differed between the viral controllers and non-controllers, we compared the total number of linear epitopes and the location of epitopes within the ZIKV polyprotein between the groups. We defined linear epitope profiles that are recognized by IgM and IgG for a subset of the dams (n= 11) following ZIKV infection using a peptide microarray.

**Figure 5:**
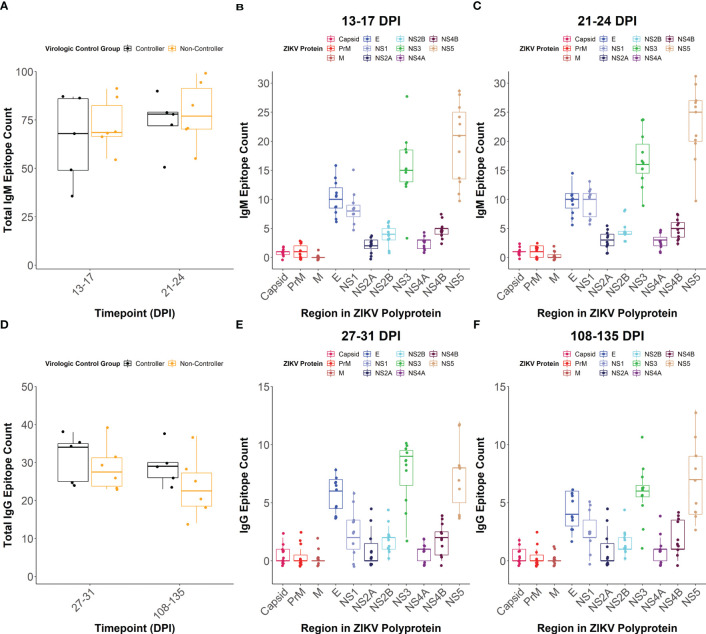
IgM and IgG linear epitope counts. The total number of linear epitopes, when defined as multiple adjacent reactive peptides, across the entire viral polyprotein was quantified at **(A)** 13-17 and 21-24 days post-infection (DPI) for IgM with dams separated based on virologic control group. The overall IgM linear epitope count within each region of the ZIKV polyprotein was also determined at **(B)** 13-17 DPI and **(C)** 21-24 DPI. For this, all dams were considered as a single population and colors correspond to individual regions of the ZIKV polyprotein. The total number of linear epitopes across the entire viral polyprotein was quantified at **(D)** 27-31 and 108-135 DPI for IgG with dams separated based on virologic control group. The total number of linear IgG epitopes within each region of the ZIKV polyprotein was determined at **(E)** 27-31 and **(F)** 108-135 DPI.

The total number of linear IgM (**Figure 5A**) and IgG (**Figure 5D**) epitopes recognized across the entire ZIKV polyprotein did not differ significantly between the viral controllers (n= 5) and non-controllers (n= 6), but the viral controllers had higher trending median epitope counts at all time points tested. There were also no significant differences in the number of IgM or IgG linear epitopes between the viral controllers and non-controllers within individual regions of the ZIKV polyprotein (**Supplementary Figure 6**). When comparing the number of linear epitopes between viral proteins for all the dams combined, Envelope (E) had a significantly higher number of total linear IgM (**Figures 5B, C**; **Supplementary Figures 7A, B**) and IgG (**Figures 5E, F**; **Supplementary Figures 7C, D**) epitopes compared to the other structural proteins (Capsid, Pre-membrane (PrM), and Membrane (M). Across the non-structural (NS) proteins, NS1, NS3, and NS5 generally had more recognized linear IgM epitopes compared to the other NS proteins (**Figures 5B, C**; **Supplementary Figures 7A, B**). In contrast, only NS3 and NS5 seemed to have more recognized linear IgG epitopes compared to the other NS proteins (**Figures 5E, F**; **Supplementary Figures 7C, D**).

In order to determine the maturation of the antibody response during pregnancy and the evolution of linear epitopes recognized by dams in the two virologic control groups, we constructed linear epitope maps for the 4 structural proteins (Capsid, PrM, M, and E) and the 7 NS proteins (NS1, NS2A, NS2B, NS3, NS4A, NS4B, and NS5) (**Supplementary Figures 8–18**). Linear epitope maps were constructed with all identified linear epitopes recognized by dams in each virologic control group, and then calculating the percentage of dams in that group that have reactivity towards individual peptides making up those linear epitopes. Overall, there were no specific epitopes in any of the viral proteins that were more commonly recognized in the viral controllers when compared to the non-controllers, or vice versa. However, there were several epitopes that were recognized by a majority of all dams regardless of their virologic control group status.

Among the structural proteins, there was a linear epitope in Capsid (peptide positions: 64-67, amino acid (AA) sequence: GLINRWGSVGKKEAMETIK) that was commonly recognized by the IgM response at 13-17 and 21-24 DPI in a majority of the dams from both virologic control groups (**Supplementary Figures 8A, B**). Additionally, there were several linear epitopes in E that were recognized by the IgG and IgM responses in dams from both virologic control groups (**Supplementary Figure 11**). Specifically, there were two linear epitopes in ectodomain (ED)II (peptide positions: 505-513, AA sequence: KEWFHDIPLPWHAGADTGTPHWNN and peptide positions: 521-526, AA sequence: TGTPHWNNKEALVEFKDAHAK) that were commonly recognized by the IgM response at 13-17 and 21-24 DPI in dams from both virologic control groups (**Supplementary Figures 11A, B**). During the IgG antibody response, a larger linear epitope was commonly recognized in EDII (peptide positions: 505-533, AA sequence: KEWFHDIPLPWHAGADTGTPHWNNKEALVEFKDAHAKRQTVVVL) at 27-31 DPI in dams from both virologic control groups and spanned the amino acids making up the two discontinuous IgM linear epitopes (**Supplementary Figure 11C**). The percentage of dams in both virologic control groups showing reactivity to this epitope decreased at 108-135 DPI (**Supplementary Figure 11D**). Additionally, there was a linear epitope in EDI (peptide positions: 450-462, AA sequence: TDENRAKVEITPNSPRAEATLGGFGSLG) that was commonly recognized by the IgG response at 27-31 and 108-135 DPI in both virologic control groups (**Supplementary Figures 11C, D**).

There were numerous IgM and IgG linear epitopes within the nonstructural proteins that were recognized by a majority of the dams. Specifically, in NS1, there were two linear epitopes, one in the wing domain (peptide positions: 832-838, AA sequence: SPRRLAAAVKQAWEDGICGISS) and one in the beta-ladder domain (peptide positions 995-1010, AA sequence: WIESEKNDTWRLKRAHLIEMKTCEWPKSHTL), that were commonly recognized by the IgM response at 13-17 and 21-24 DPI in dams from both virologic control groups (**Supplementary Figures 12A, B**). During the IgG antibody response, shared reactivity to these epitopes decreased and fewer epitopes were recognized overall in both virologic control groups (**Supplementary Figures 12C, D**). In NS2B there was a linear epitope (peptide positions 1382-1389, AA sequence: AVGLICALAGGFAKADIEMAGPM) that was commonly recognized by the IgM response at 13-17 and 21-24 DPI in dams from both virologic control groups, as well as a second linear epitope (peptide position: 1409-1416, AA sequence: LLIVSYVVSGKSVDMYIERAGDI) at 21-24 DPI (**Supplementary Figures 14A, B**). This differed from what was seen during the IgG response at 27-31 and 108-135 DPI where a single, large, linear epitope (peptide position 1430-1452, AA sequence: DITWEKDAEVTGNSPRLDVALDESGDFSLVEDDGPPM) was recognized in dams from both virologic control groups (**Supplementary Figures 14C, D**), similar to what has been previously reported (52).

## 4 Discussion

Here, we provide the first in-depth analysis of the magnitude and breadth of the maternal antibody response following ZIKV infection in pregnant macaques. We found that maternal virologic control may be determined within the first 7 DPI and was characterized by shorter plasma vRNA burden duration and lower vRNA burden in the MFI at delivery. Additionally, we show that a higher magnitude and greater breadth of the maternal ZIKV-specific antibody response was not associated with better maternal virologic control. We identified increased IgM and IgG ZIKV-specific antibody titers throughout pregnancy in the viral non-controllers, which could serve as a biomarker to identify mothers with worse virologic control. We focused specifically on antibody responses here, leaving room for the examination of other host factors that may be responsible for differential viral control in future studies. Different T cell responses, innate antiviral responses, and major histocompatibility complex genotypes should be evaluated in future studies to help explain the wide spectrum of clinical phenotypes observed in human congenital ZIKV infection, ranging from asymptomatic to developmental deficits to severe birth defects.

Viral non-controllers had prolonged plasma vRNA burden and had a higher vRNA burden in the MFI tissues at delivery. The appearance of prolonged plasma vRNA burden in our study corroborates the findings of other NHP studies and human cases of ZIKV infection during pregnancy (15–19, 41, 42, 56, 57). Since prolonged plasma vRNA burden is never observed during ZIKV infection of non-pregnant NHPs, there is likely a pregnancy-specific reservoir of viral replication that may be the source of prolonged vRNA shedding into the maternal blood (36, 42, 58, 59). Although some viral non-controllers exhibited prolonged plasma vRNA burden, we only detected infectious virus in the plasma after 7 DPI in one dam (044–133). This suggests that prolonged vRNA detection in the maternal blood of several non-controller dams may be a result of focal viral replication in a specific site that sheds vRNA into the blood, but does not shed infectious virus that we are able to detect in a plaque assay. Higher vRNA burdens at the time of delivery in the immune-privileged MFI tissues of the viral non-controllers compared to the controllers indicates that this may be the pregnancy-specific site of focal ZIKV replication that is leading to the prolonged vRNA shedding that we observe. We hypothesize that in the viral non-controllers ZIKV reaches this site by 7 DPI, when infectious virus is still detectable in the maternal blood, and that establishment of this viral reservoir leads to the prolonged plasma vRNA burden and increased vRNA burden in the MFI that is observed. This is consistent with a recent report that ZIKV is able to reach the MFI tissues as early as 7 DPI (60). Maternal plasma vRNA burden was cleared prior to delivery in all dams regardless of the level of vRNA burden in the MFI tissues at delivery. This may be because virus is replicating at very low levels up until delivery in the MFI and the vRNA in the plasma exists at low titers that are undetectable via RT-qPCR, or it may be a result of infectious virus being cleared from the MFI tissues prior to delivery and the vRNA we are detecting in the MFI is residual from prior infection. It should be noted that one limitation to our study is that we were only able to assess vRNA in the MFI at the time of delivery as all pregnancies proceeded to term. Thus, the absence of vRNA in the MFI at delivery does not preclude the possibility that ZIKV reached those tissues at some point during pregnancy and was effectively cleared before delivery. However, the detection of vRNA in the MFI at delivery is likely indicative of more extensive and prolonged viral replication in these tissues, which would be characteristic of worse virologic control. Overall, we have shown that prolonged plasma vRNA burden and viral dissemination to the MFI are key characteristics of poor control of ZIKV infection during pregnancy. Establishment of a viral reservoir in the MFI may be the source of prolonged plasma vRNA burden seen in some dams, and it also seems that the viral reservoir may be established in the first 7 DPI.

Viral non-controllers had higher peak plasma vRNA titers compared to viral controllers, but their peak infectious virus titers did not differ. An earlier study in non-pregnant macaques found that detectable infectious virus titers were ~500-1,000 fold lower than vRNA loads (42). We show that in pregnant macaques this could be more variable with detectable infectious virus titers being anywhere from ~100-2,300 fold lower than vRNA loads on the day of peak plasma vRNA burden. Interestingly, the viral non-controllers had vRNA to infectious virus ratios that trended higher, which indicates that these dams may have increased levels of circulating vRNA but do not have proportionately increased levels of detectable infectious virus. This suggests that the higher vRNA titers found early in infection in the viral non-controllers may exist in a form that we are unable to detect in a plaque assay. ZIKV vRNA and proteins can be incorporated into cell-derived extracellular vesicles that exhibit variable infectivity in cell culture (61, 62). Thus, it is possible that vRNA packaged in extracellular vesicles is leading to higher vRNA loads early in infection in the viral non-controllers. The contents of extracellular vesicles (vRNA and protein) may serve a pathogenic role in ZIKV infection during pregnancy and may play an important role in determining maternal virologic control. Future work should focus on understanding the nature of extracellular vesicles containing ZIKV vRNA and proteins, the role they play in pathogenesis during pregnancy, and how they impact maternal virologic control.

Our results also highlighted higher maternal ZIKV-specific antibody titers in viral non-controllers supporting that this feature could serve as a biomarker to identify mothers with worse control of ZIKV infection. We consistently found higher ZIKV-specific binding and neutralizing antibody titers in the viral non-controllers over the course of pregnancy. In contrast, the breadth of the linear epitope responses did not differ between the viral controllers and non-controllers. A key limitation to our data is that we do not examine the antibody response to tertiary or quaternary epitope structures, which could also be important for maternal virologic control and may differ between the groups (63). Similar to our findings, data from a cohort in Brazil found that higher maternal ZIKV-specific neutralizing antibody titers at delivery were associated with cases of microcephaly in ZIKV-exposed infants (64). The association of higher antibody titers in cases of poor maternal virologic control and worse infant outcomes may be due to IgG binding to ZIKV and facilitating transplacental transmission via the neonatal Fc-receptor, which has been shown to occur in the presence of cross-reactive DENV antibodies (65). However, this is unlikely in our model as all dams are flavivirus-naive and it is unlikely that ZIKV-specific IgG antibodies would bind weakly enough to facilitate transplacental transmission without neutralizing the virus. Additionally, higher IgG antibody titers are not observed in the viral non-controllers until 3-4 weeks post-infection, which is beyond 7 DPI when we believe maternal virologic control is being established. We would like to make a careful point that our studies describe the antibody titers in response to infection, and not the antibody responses required to prevent infection, because these are all flavivirus naïve animals. We believe that higher antibody titers in the viral non-controllers is a by-product of poor virologic control, rather than a factor contributing to it. Thus, we believe increased antibody titers could be used as a biomarker to identify human cases of poor maternal infection control. This should be evaluated in ZIKV pregnancy human cohort studies, using serologic assays that better distinguish DENV and ZIKV antibody titers.

Overall, our cohort of ZIKV-infected pregnant macaques yielded several important insights related to maternal infection control that are translatable to humans. The first seven days following inoculation are a critical period where maternal infection control is likely determined, and when it has been shown that ZIKV has already reached the MFI (60). Therefore, future interventional studies need to act within this early period to increase the chances of successfully controlling maternal infection. Because early identification of ZIKV infection is challenging when women are asymptomatic or have non-specific signs of infection, vaccination of at-risk populations is key. Additionally, poor maternal infection control may be related to particles with variable levels of infectivity in a plaque assay, such as extracellular vesicles that contain ZIKV vRNA and proteins, given the discrepancy between peak vRNA and detectable infectious virus titers. Future studies evaluating the potential role of these particles in disease pathogenesis during maternal ZIKV infection is critical for developing novel therapeutics. Finally, higher ZIKV-specific binding and neutralizing antibody titers were a biomarker of worse maternal infection control, and these markers should be evaluated in clinical studies to determine whether they can assist in the prognosis of pregnancy and infant outcomes. Maternal ZIKV infection can lead to severe birth defects and neurodevelopmental deficits in offspring: evaluating these potential new biomarkers and markers of pathogenesis in clinical studies may help improve child health globally.

## Data availability statement

The datasets presented in this study can be found in online repositories. The names of the repository/repositories and accession number(s) can be found below: https://go.wisc.edu/pzl09d.

## Ethics statement

The animal study was approved by University of Wisconsin-Madison Institutional Animal Care and Use Committee. The study was conducted in accordance with the local legislation and institutional requirements.

## Author contributions

NK: Data curation, Formal Analysis, Investigation, Methodology, Visualization, Writing – original draft, Writing – review & editing, Conceptualization. ER: Writing – review & editing. HA: Conceptualization, Investigation, Writing – review & editing. RS: Investigation, Writing – review & editing. YS: Investigation, Writing – review & editing. SB: Writing – review & editing. EB: Investigation, Writing – review & editing. JP: Investigation, Writing – review & editing. AW: Investigation, Writing – review & editing. AM: Investigation, Writing – review & editing. JE: Formal Analysis, Writing – review & editing. ES: Investigation, Resources, Writing – review & editing. JT: Resources, Supervision, Writing – review & editing. MA: Supervision, Writing – review & editing. TF: Supervision, Writing – review & editing. DO: Funding acquisition, Project administration, Writing – review & editing. TG: Funding acquisition, Project administration, Supervision, Writing – review & editing. EM: Conceptualization, Funding acquisition, Methodology, Project administration, Resources, Supervision, Validation, Writing – original draft, Writing – review & editing.
