# Supplementary material for: Control of maternal Zika virus infection during pregnancy is associated with lower antibody titers in a macaque model
Source: Front Immunol. 2023 Sep 22;14:1267638. doi: 10.3389/fimmu.2023.1267638 (PMC10556460; doi:10.3389/fimmu.2023.1267638)
Supplement: Supplementary file 1 [file DataSheet_1.docx]

Supplementary Material

# Supplementary Figures and Tables

## Supplementary Tables

**Supplementary Table 1. Specific timepoints of each antibody assay for individual dams**. “NT” = not tested, “DPI” = days post-infection.

| **Dam ID** | **Assay** | **Pre-**  **Infection** | **2-4**  **DPI** | **7-10**  **DPI** | **13-17**  **DPI** | **18-24**  **DPI** | **27-38**  **DPI** | **52-66**  **DPI** | **84-94**  **DPI** | **98-117**  **DPI** | **121-136**  **DPI** |
| --- | --- | --- | --- | --- | --- | --- | --- | --- | --- | --- | --- |
| 044-101 | IgM Binding ELISA | 0 DPI | 2 DPI | 10 DPI | 14 DPI | 21 DPI | 28 DPI | 56 DPI | 84 DPI | NT | NT |
|  | IgM Peptide Array | NT | NT | NT | 17 DPI | 21 DPI | NT | NT | NT | NT | NT |
|  | IgG Whole-Virion Binding ELISA | 0 DPI | 2 DPI | 8 DPI | 17 DPI | NT | 28 DPI | 56 DPI | 94 DPI | 112 DPI | NT |
|  | PRNT | 0 DPI | NT | NT | NT | NT | 28 DPI | NT | NT | 101 DPI | NT |
|  | IgG Peptide Array | NT | NT | NT | NT | NT | 28 DPI | NT | NT | 112 DPI | NT |
| 044-102 | IgM Binding ELISA | 0 DPI | 4 DPI | 10 DPI | 15 DPI | 21 DPI | 28 DPI | 59 DPI | NT | NT | NT |
|  | IgM Peptide Array | NT | NT | NT | 15 DPI | 24 DPI | NT | NT | NT | NT | NT |
|  | IgG Whole-Virion Binding ELISA | 0 DPI | 4 DPI | 7 DPI | 15 DPI | 24 DPI | 28 DPI | 66 DPI | 91 DPI | 115 DPI | NT |
|  | PRNT | 0 DPI | NT | NT | NT | NT | 28 DPI | NT | NT | 101 DPI | NT |
|  | IgG Peptide Array | NT | NT | NT | NT | NT | 28 DPI | NT | NT | 115 DPI | NT |
| 044-103 | IgM Binding ELISA | NT | NT | 10 DPI | 15 DPI | 22 DPI | 35 DPI | NT | NT | NT | NT |
|  | IgM Peptide Array | NT | NT | NT | 15 DPI | 22 DPI | NT | NT | NT | NT | NT |
|  | IgG Whole-Virion Binding ELISA | -6 DPI | 4 DPI | 7 DPI | 15 DPI | 22 DPI | 35 DPI | 52 DPI | 87 DPI | 114 DPI | NT |
|  | PRNT | 0 DPI | NT | NT | NT | NT | 35 DPI | NT | NT | 98 DPI | NT |
|  | IgG Peptide Array | NT | NT | NT | NT | NT | 31 DPI | NT | NT | 114 DPI | NT |
| 044-104 | IgM Binding ELISA | NT | NT | 10 DPI | 15 DPI | 18 DPI | 29 DPI | 59 DPI | NT | NT | NT |
|  | IgM Peptide Array | NT | NT | NT | 15 DPI | 22 DPI | NT | NT | NT | NT | NT |
|  | IgG Whole-Virion Binding ELISA | NT | NT | 10 DPI | 15 DPI | 22 DPI | 29 DPI | 59 DPI | 87 DPI | 114 DPI | NT |
|  | PRNT | 0 DPI | NT | NT | NT | NT | 35 DPI | NT | NT | 105 DPI | NT |
|  | IgG Peptide Array | NT | NT | NT | NT | NT | 29 DPI | NT | NT | 114 DPI | NT |
| 044-109 | IgM Binding ELISA | 0 DPI | 4 DPI | 10 DPI | 14 DPI | 21 DPI | 28 DPI | 59 DPI | NT | NT | NT |
|  | IgM Peptide Array | NT | NT | NT | 14 DPI | 21 DPI | NT | NT | NT | NT | NT |
|  | IgG Whole-Virion Binding ELISA | 0 DPI | 4 DPI | 7 DPI | 14 DPI | 21 DPI | 28 DPI | 59 DPI | 87 DPI | 108 DPI | NT |
|  | PRNT | 0 DPI | NT | NT | NT | NT | 28 DPI | NT | NT | 108 DPI | NT |
|  | IgG Peptide Array | NT | NT | NT | NT | NT | 28 DPI | NT | NT | 108 DPI | NT |
| 044-110 | IgM Binding ELISA | 0 DPI | 4 DPI | 10 DPI | 13 DPI | 20 DPI | 27 DPI | NT | NT | NT | NT |
|  | IgM Peptide Array | NT | NT | NT | 13 DPI | 23 DPI | NT | NT | NT | NT | NT |
|  | IgG Whole-Virion Binding ELISA | 0 DPI | 4 DPI | 7 DPI | 13 DPI | 23 DPI | 27 DPI | 58 DPI | 86 DPI | 114 DPI | 132 DPI |
|  | PRNT | 0 DPI | NT | NT | NT | NT | 27 DPI | NT | NT | 114 DPI | NT |
|  | IgG Peptide Array | NT | NT | NT | NT | NT | 27 DPI | NT | NT | NT | 132 DPI |
| 044-112 | IgM Binding ELISA | NT | 4 DPI | 10 DPI | 16 DPI | 20 DPI | 27 DPI | 58 DPI | 86 DPI | NT | NT |
|  | IgM Peptide Array | 0 DPI | NT | NT | 16 DPI | 23 DPI | NT | NT | NT | NT | NT |
|  | IgG Whole-Virion Binding ELISA | 0 DPI | 4 DPI | 7 DPI | 16 DPI | 23 DPI | 27 DPI | 58 DPI | 86 DPI | 114 DPI | 121 DPI |
|  | PRNT | 0 DPI | NT | NT | NT | NT | 27 DPI | NT | NT | 114 DPI | NT |
|  | IgG Peptide Array | NT | NT | NT | NT | NT | 27 DPI | NT | NT | NT | 129 DPI |
| 044-114 | IgM Binding ELISA | 0 DPI | 4 DPI | 10 DPI | 14 DPI | 21 DPI | 28 DPI | 59 DPI | NT | NT | NT |
|  | IgM Peptide Array | NT | NT | NT | 14 DPI | 21 DPI | NT | NT | NT | NT | NT |
|  | IgG Whole-Virion Binding ELISA | 0 DPI | 4 DPI | 7 DPI | 14 DPI | 21 DPI | 28 DPI | 59 DPI | 87 DPI | 108 DPI | 135 DPI |
|  | PRNT | 0 DPI | NT | NT | NT | NT | 28 DPI | NT | NT | 108 DPI | NT |
|  | IgG Peptide Array | NT | NT | NT | NT | NT | 28 DPI | NT | NT | NT | 135 DPI |
| 044-116 | IgM Binding ELISA | 0 DPI | 4 DPI | 7 DPI | 13 DPI | 20 DPI | 30 DPI | NT | NT | NT | NT |
|  | IgM Peptide Array | NT | NT | NT | 13 DPI | 22 DPI | NT | NT | NT | NT | NT |
|  | IgG Whole-Virion Binding ELISA | 0 DPI | 4 DPI | 7 DPI | 13 DPI | 23 DPI | 30 DPI | 58 DPI | 86 DPI | NT | 135 DPI |
|  | PRNT | 0 DPI | NT | NT | NT | NT | 30 DPI | NT | NT | NT | 135 DPI |
|  | IgG Peptide Array | NT | NT | NT | NT | NT | 29 DPI | NT | NT | NT | 134 DPI |
| 044-117 | IgM Binding ELISA | 0 DPI | 4 DPI | 8 DPI | 15 DPI | 22 DPI | 33 DPI | NT | NT | NT | NT |
|  | IgM Peptide Array | NT | NT | NT | 15 DPI | 22 DPI | NT | NT | NT | NT | NT |
|  | IgG Whole-Virion Binding ELISA | 0 DPI | 4 DPI | 8 DPI | 15 DPI | 22 DPI | 33 DPI | NT | NT | NT | 125 DPI |
|  | PRNT | 0 DPI | NT | NT | NT | NT | 30 DPI | NT | NT | NT | 125 DPI |
|  | IgG Peptide Array | NT | NT | NT | NT | NT | 29 DPI | NT | NT | NT | 130 DPI |
| 044-118 | IgM Binding ELISA | 0 DPI | 4 DPI | 10 DPI | 15 DPI | 18 DPI | 29 DPI | 53 DPI | NT | NT | NT |
|  | IgM Peptide Array | NTI | NT | NT | 16 DPI | 23 DPI | NT | NT | NT | NT | NT |
|  | IgG Whole-Virion Binding ELISA | 0 DPI | 4 DPI | 7 DPI | 15 DPI | 18 DPI | 29 DPI | 53 DPI | NT | 116 DPI | 123 DPI |
|  | PRNT | 0 DPI | NT | NT | NT | NT | 29 DPI | NT | NT | NT | 123 DPI |
|  | IgG Peptide Array | NT | NT | NT | NT | NT | 28 DPI | NT | NT | NT | 126 DPI |
| 044-122 | IgM Binding ELISA | 0 DPI | 4 DPI | 10 DPI | 14 DPI | 21 DPI | 32 DPI | 53 DPI | 88 DPI | NT | NT |
|  | IgM Peptide Array | NT | NT | NT | NT | NT | NT | NT | NT | NT | NT |
|  | IgG Whole-Virion Binding ELISA | 0 DPI | 4 DPI | 8 DPI | 14 DPI | 21 DPI | 32 DPI | 53 DPI | 88 DPI | 116 DPI | 123 DPI |
|  | PRNT | 0 DPI | NT | NT | NT | NT | 32 DPI | NT | NT | NT | 123 DPI |
|  | IgG Peptide Array | NT | NT | NT | NT | NT | NT | NT | NT | NT | NT |
| 044-126 | IgM Binding ELISA | 0 DPI | 4 DPI | 10 DPI | 14 DPI | 18 DPI | 28 DPI | 53 DPI | 88 DPI | NT | NT |
|  | IgM Peptide Array | NT | NT | NT | NT | NT | NT | NT | NT | NT | NT |
|  | IgG Whole-Virion Binding ELISA | 0 DPI | 4 DPI | 7 DPI | 14 DPI | 18 DPI | 28 DPI | 53 DPI | 88 DPI | 117 DPI | NT |
|  | PRNT | 0 DPI | NT | NT | NT | NT | 32 DPI | NT | NT | 117 DPI | NT |
|  | IgG Peptide Array | NT | NT | NT | NT | NT | NT | NT | NT | NT | NT |
| 044-127 | IgM Binding ELISA | 0 DPI | 2 DPI | 8 DPI | 14 DPI | 21 DPI | 31 DPI | 52 DPI | 91 DPI | NT | NT |
|  | IgM Peptide Array | NT | NT | NT | NT | NT | NT | NT | NT | NT | NT |
|  | IgG Whole-Virion Binding ELISA | 0 DPI | 2 DPI | 8 DPI | 14 DPI | 21 DPI | 31 DPI | 52 DPI | 91 DPI | 112 DPI | NT |
|  | PRNT | 0 DPI | NT | NT | NT | NT | 38 DPI | NT | NT | 112 DPI | NT |
|  | IgG Peptide Array | NT | NT | NT | NT | NT | NT | NT | NT | NT | NT |
| 044-130 | IgM Binding ELISA | 0 DPI | 4 DPI | 7 DPI | 14 DPI | 21 DPI | 31 DPI | NT | NT | NT | NT |
|  | IgM Peptide Array | NT | NT | NT | NT | NT | NT | NT | NT | NT | NT |
|  | IgG Whole-Virion Binding ELISA | 0 DPI | 4 DPI | 7 DPI | 14 DPI | 21 DPI | 31 DPI | 56 DPI | 91 DPI | 112 DPI | NT |
|  | PRNT | 0 DPI | NT | NT | NT | NT | 31 DPI | NT | NT | 112 DPI | NT |
|  | IgG Peptide Array | NT | NT | NT | NT | NT | NT | NT | NT | NT | NT |
| 044-131 | IgM Binding ELISA | 0 DPI | 4 DPI | 7 DPI | 13 DPI | 20 DPI | 31 DPI | 55 DPI | 90 DPI | NT | NT |
|  | IgM Peptide Array | NT | NT | NT | NT | NT | NT | NT | NT | NT | NT |
|  | IgG Whole-Virion Binding ELISA | 0 DPI | 4 DPI | 7 DPI | 13 DPI | 20 DPI | 31 DPI | 55 DPI | 90 DPI | 115 DPI | 136 DPI |
|  | PRNT | 0 DPI | NT | NT | NT | NT | 31 DPI | NT | NT | NT | 136 DPI |
|  | IgG Peptide Array | NT | NT | NT | NT | NT | NT | NT | NT | NT | NT |
| 044-132 | IgM Binding ELISA | 0 DPI | 4 DPI | 7 DPI | 14 DPI | 21 DPI | 31 DPI | NT | NT | NT | NT |
|  | IgM Peptide Array | NT | NT | NT | NT | NT | NT | NT | NT | NT | NT |
|  | IgG Whole-Virion Binding ELISA | 0 DPI | 4 DPI | 7 DPI | 14 DPI | 21 DPI | 31 DPI | 56 DPI | 91 DPI | 115 DPI | 129 DPI |
|  | PRNT | 0 DPI | NT | NT | NT | NT | 31 DPI | NT | NT | NT | 129 DPI |
|  | IgG Peptide Array | NT | NT | NT | NT | NT | NT | NT | NT | NT | NT |
| 044-133 | IgM Binding ELISA | 0 DPI | 2 DPI | 8 DPI | 15 DPI | 22 DPI | 29 DPI | NT | NT | NT | NT |
|  | IgM Peptide Array | NT | NT | NT | NT | NT | NT | NT | NT | NT | NT |
|  | IgG Whole-Virion Binding ELISA | 0 DPI | 2 DPI | 8 DPI | 15 DPI | 22 DPI | 29 DPI | 57 DPI | 92 DPI | 117 DPI | NT |
|  | PRNT | 0 DPI | NT | NT | NT | NT | 31 DPI | NT | NT | 117 DPI | NT |
|  | IgG Peptide Array | NT | NT | NT | NT | NT | NT | NT | NT | NT | NT |

**Supplementary Table 2. Commercial antibodies used in serological assays.**

| **Reagent** | **Assay** | **Manufacturer** | **Clone** | **Product number** |
| --- | --- | --- | --- | --- |
| Mouse Anti-Monkey IgG-Alexa Fluor® 647 | IgG Peptide Array | Southern  Biotech | SB108a | 4700-31 |
| Goat Anti-Monkey IgM (μ-chain specific)-Rhodamine antibody | IgM Peptide Array | Millipore Sigma | polyclonal | SAB3700777 |
| Recombinant Anti-Flavivirus E-glycoprotein antibody [D1-4G2-4-15 (4G2)] | IgG Whole-Virion Binding ELISA | Absolute Antibody | Monoclonal | Ab00230-10.0 |
| Mouse Anti-Monkey IgG-HRP | IgG Whole-Virion Binding ELISA | Southern  Biotech | SB108a | 4700-05 |

**Supplementary Table 3. Amino acid positions of individual ZIKV (PRVABC59) viral proteins within the full-length polyprotein (GenBank: KU501215 (nucleotide sequence)/AMC13911 (amino acid sequence)).** Amino acid positions were determined based on the Virus Pathogen Database and Analysis Resource (ViPR) (1). “EDI” = envelope ectodomain I, “EDII” = envelope ectodomain II, “EDIII” = envelope ectodomain III, “TM" = envelope transmembrane domain.

| **ZIKV Protein** | **Amino acid position** |
| --- | --- |
| Capsid | 1-122 |
| Premembrane | 123-215 |
| Membrane | 216-290 |
| Envelope | 291-794 |
| EDI | 291-341, 422-482, 570-585 |
| EDII | 342-421, 483-569 |
| EDIII | 586-693 |
| TM | 698-794 |
| NS1 | 795-1146 |
| Beta-roll | 795-825 |
| Wing | 826-976 |
| Beta-ladder | 977-1146 |
| NS2A | 1147-1372 |
| NS2B | 1373-1502 |
| NS3 | 1503-2119 |
| NS4A | 2120-2269 |
| NS4B | 2270-2520 |
| NS5 | 2521-3423 |

**Supplementary Table 4. Latent class analysis (LCA) clustering results.** “High” and “Low” duration of plasma vRNA burden correspond to ≥8 days post-infection (DPI) or <8 DPI, respectively. “High” and “Low” total maternal-fetal interface (MFI) biopsies vRNA-positive (%) correspond to ≥4% and <4%, respectively. “High” and “Low” area under the curve (AUC) values correspond to ≥108,609 and <108,609, respectively.  “NA” = dam gave birth naturally and MFI tissues were unable to be collected.

| **Dam ID** | **Viral parameters used for LCA** | | | **Designation of viral parameters from LCA** | | | **Virologic Control Group Designation** | **Probability of Non-**  **Controller Group Designation (%)** | **Probability of Controller Group Designation (%)** |
| --- | --- | --- | --- | --- | --- | --- | --- | --- | --- |
|  | **Duration of Plasma vRNA Burden (DPI)** | **Total MFI Biopsies vRNA-Positive (%)** | **AUC Values** | **Duration of Plasma vRNA Burden (DPI)** | **Total MFI Biopsies vRNA-Positive (%)** | **AUC Values** |  |  |  |
| 044-101 | 31 | 25 | 1288522 | High | High | High | Non-  Controller | 100 | 0 |
| 044-102 | 6 | 2 | 7367.31 | Low | Low | Low | Controller | 0 | 100 |
| 044-103 | 6 | 0 | 8933.24 | Low | Low | Low | Controller | 0 | 100 |
| 044-104 | 8 | 3 | 153302.4 | High | Low | High | Non-  Controller | 98 | 2 |
| 044-109 | 9 | NA | 30968.65 | High | NA | Low | Non-  Controller | 82 | 18 |
| 044-110 | 5 | 0 | 17127.34 | Low | Low | Low | Controller | 0 | 100 |
| 044-112 | 10 | 8 | 461954 | High | High | High | Non-  Controller | 100 | 0 |
| 044-114 | 28 | 4 | 290702 | High | High | High | Non-  Controller | 100 | 0 |
| 044-116 | 7 | 0 | 8299.66 | Low | Low | Low | Controller | 0 | 100 |
| 044-117 | 6 | 0 | 141388.2 | Low | Low | High | Controller | 0 | 100 |
| 044-118 | 10 | 4 | 696723.2 | High | High | High | Non-  Controller | 100 | 0 |
| 044-122 | 52 | 5 | 144474.9 | High | High | High | Non-  Controller | 100 | 0 |
| 044-126 | 45 | 11 | 801903.7 | High | High | High | Non-  Controller | 100 | 0 |
| 044-127 | 4 | 0 | 62932.9 | Low | Low | Low | Controller | 0 | 100 |
| 044-130 | 7 | 14 | 75830.25 | Low | High | Low | Controller | 0 | 100 |
| 044-131 | 7 | NA | 15111.25 | Low | NA | Low | Controller | 0 | 100 |
| 044-132 | 5 | 0 | 9436.2 | Low | Low | Low | Controller | 0 | 100 |
| 044-133 | 29 | 6 | 358270 | High | High | High | Non-  Controller | 100 | 0 |

## Supplementary Figures


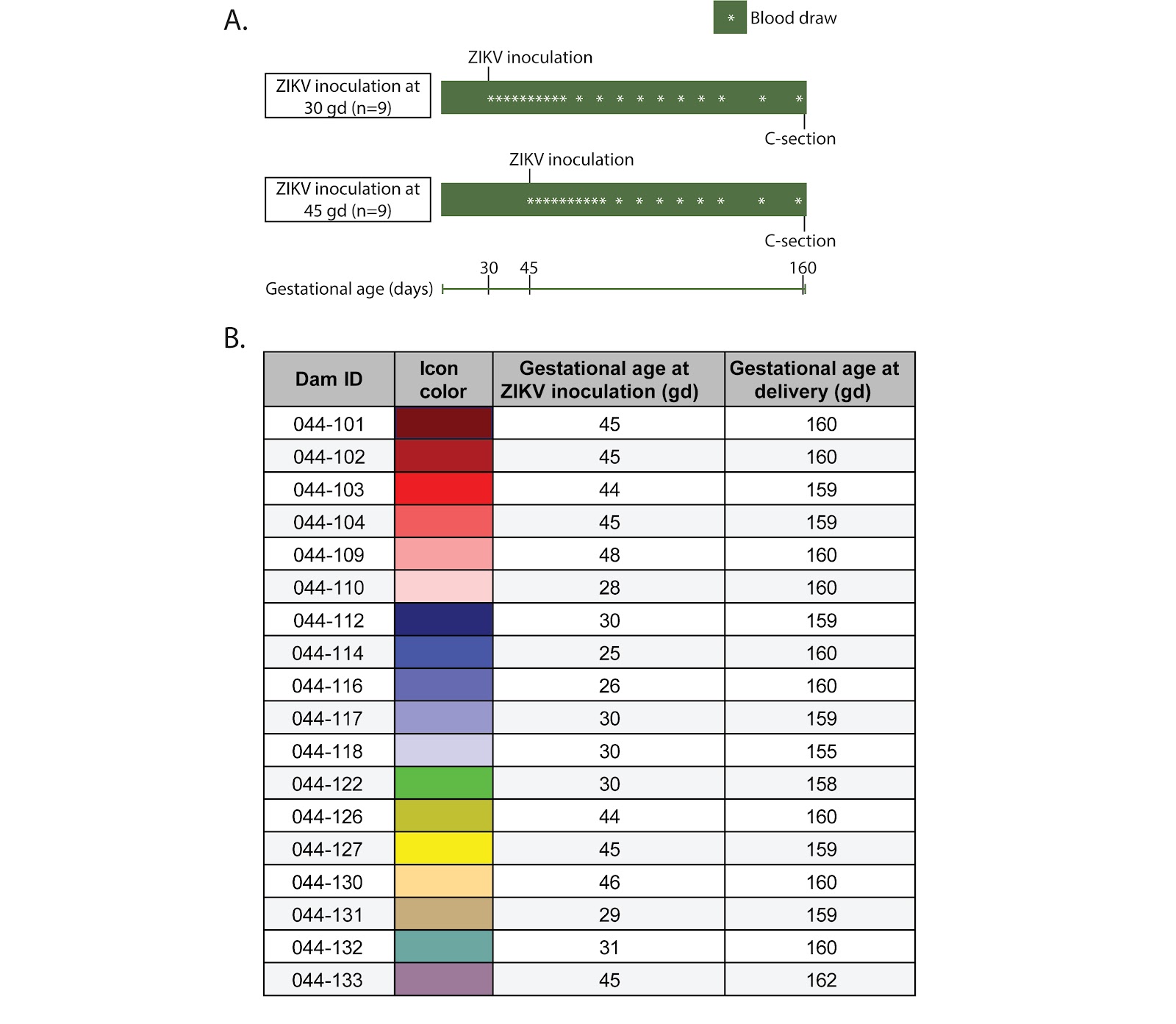


**Supplementary Figure 1. Experimental design and key time points for individual animals.** **(A)** Experimental design showing the approximate time point of ZIKV inoculation and delivery. Approximate timing of blood draws during pregnancy are indicated. **(B)** Specific gestational ages of ZIKV inoculation and delivery for each animal. “gd” = gestational days.


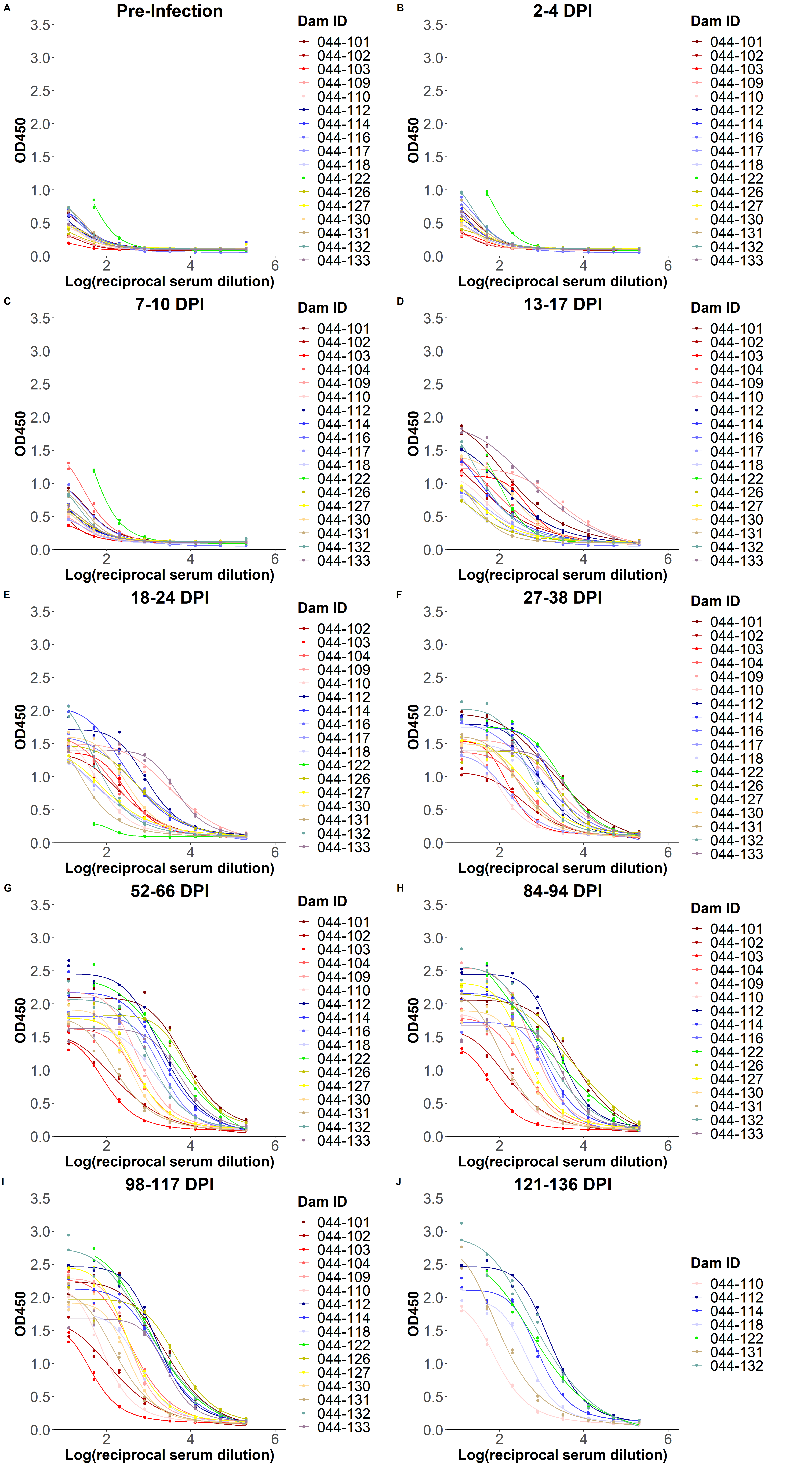


**Supplementary Figure 2. ZIKV whole-virion IgG binding antibody assay raw curves**. Optical density readings were performed at 450nm (OD450) over a range of serum dilutions for dams at **(A)** pre-infection, **(B)** 2-4 DPI, **(C)** 7-10 DPI, **(D)** 13-17 DPI, **(E)** 18-24 DPI, **(F)** 27-38 DPI, **(G)** 52-66 DPI, **(H)** 84-94 DPI, **(I)** 98-117 DPI, and **(J)** 121-136 DPI. The resulting data was plotted using R statistical language (R Core Team 2022), version 4.2.2, to generate raw IgG binding antibody curves for each dam at each timepoint.


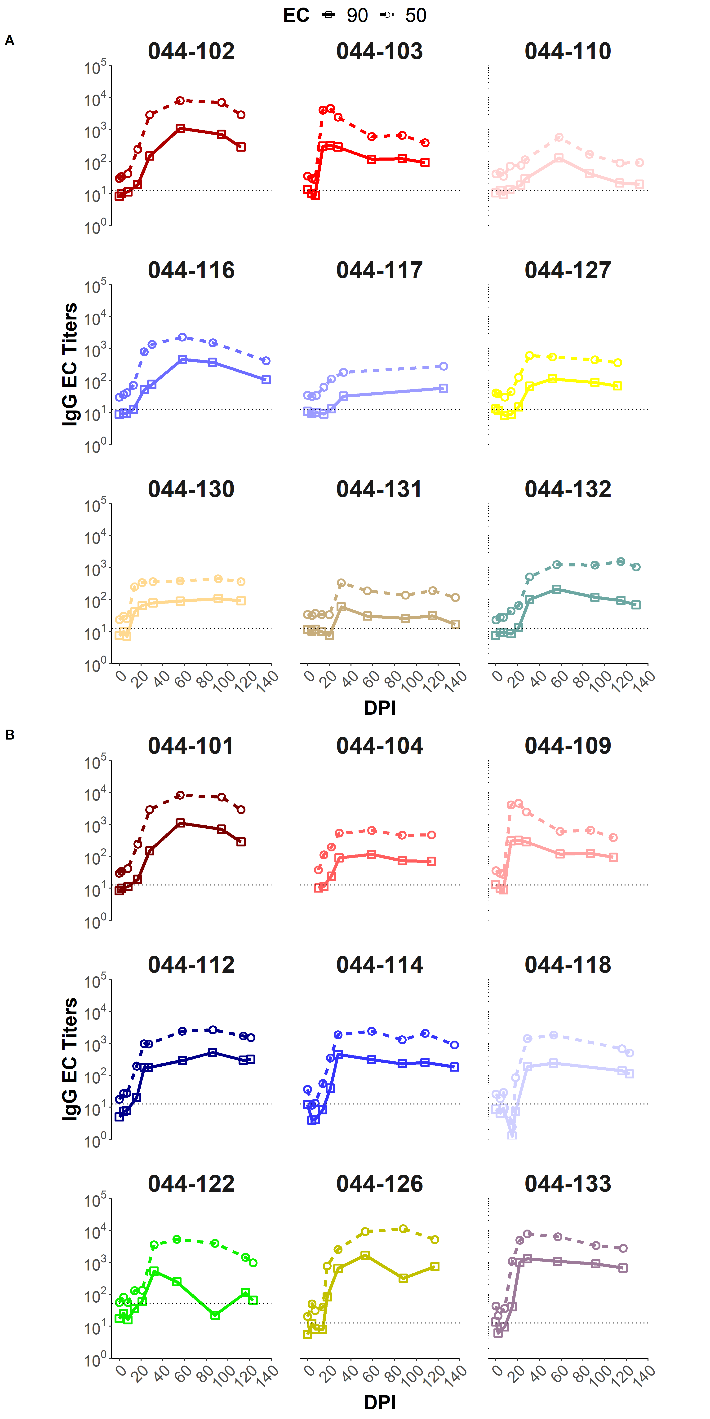


**Supplementary Figure 3. ZIKV-specific IgG binding EC_90_ and E_50_ titers over time for each dam**. The R extension package ‘drc’ (50) was used to generate 4-parameter dose-response models based on the raw IgG binding antibody curves (**Supplementary Figure 2**). The effective dilution of serum to reduce the maximum OD450 reading by 90% and 50% (EC_90_ (solid line) and EC_50_ (dashed line), respectively) were estimated for each dam at each timepoint tested based on the dose-response models. Individual dams were separated based on their classification as a **(A)** controller or **(B)** non-controller. The limit of detection for each dam is indicated by a dotted line on the figure.


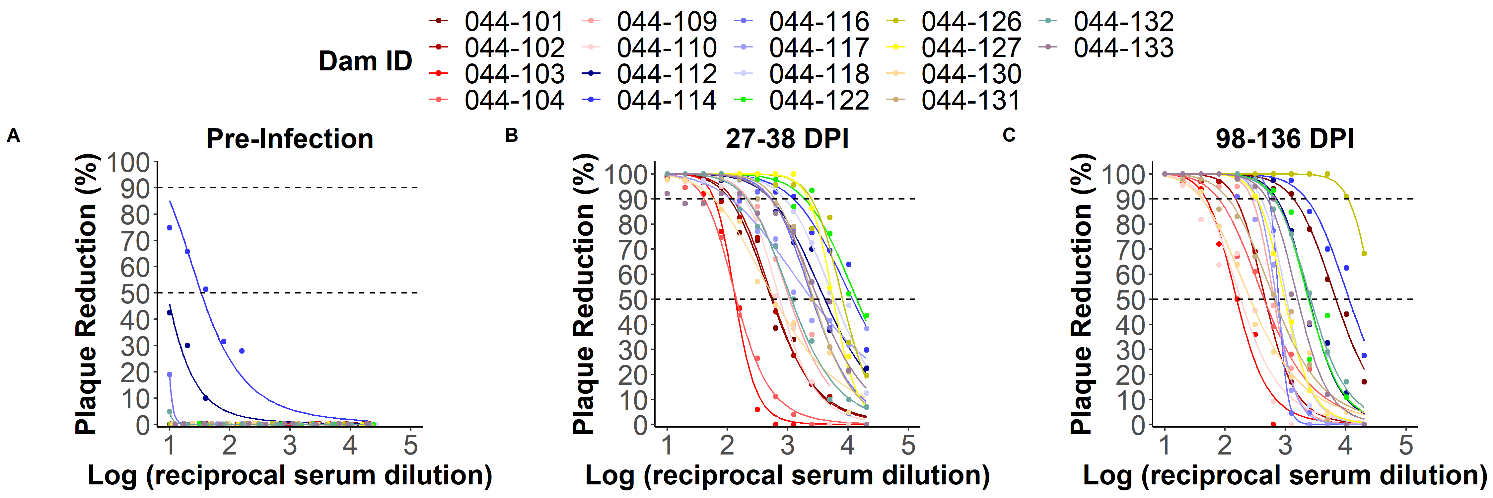


**Supplementary Figure 4. Raw ZIKV plaque reduction neutralization curves**. Percent plaque reduction was determined over a range of serum dilutions for dams at **(A)** pre-infection, **(B)** 27-38 DPI, and **(C)** 98-136 DPI. The resulting data was plotted using R statistical language (R Core Team 2022), version 4.2.2, to generate raw ZIKV neutralization curves for each dam at each time point. The dashed lines indicate 90% plaque reduction and 50% plaque reduction.


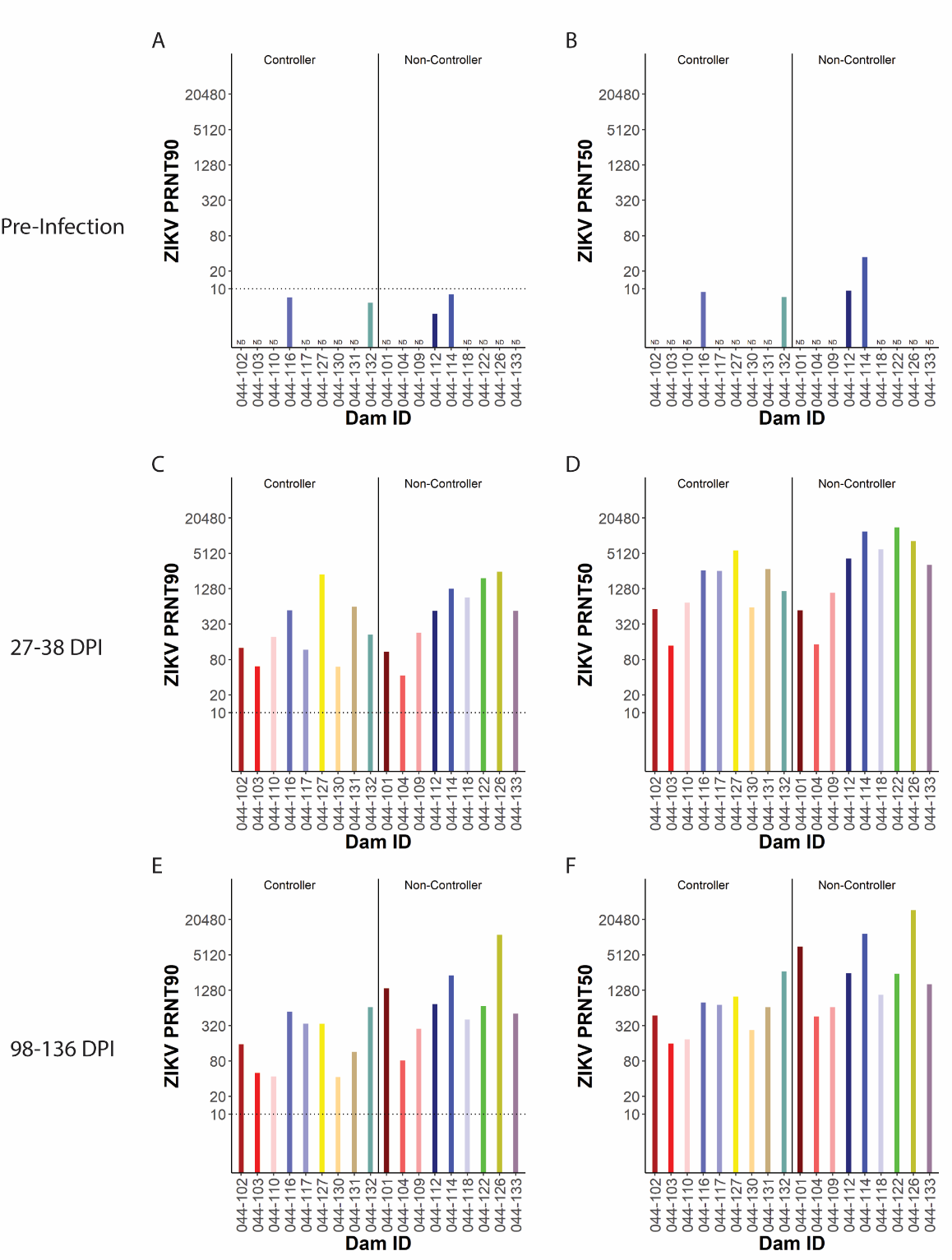


**Supplementary Figure 5. ZIKV-specific neutralizing antibody titers for each dam**. The R extension package ‘drc’ (50) was used to generate 4-parameter dose-response models based on the raw ZIKV plaque reduction neutralization curves (**Supplementary Figure 4**). The dilution of serum to reduce the number of plaques by 90% and 50% (PRNT_90_ and PRNT_50_, respectively) was estimated for each dam at **(A-B)** pre-infection, **(C-D)** 27-38 DPI, and **(E-F)** 98-136 DPI. Individual dams are grouped based on their classification as a controller or non-controller. The limit of detection for the assay is indicated by a dotted line on the PRNT_90_ figures.


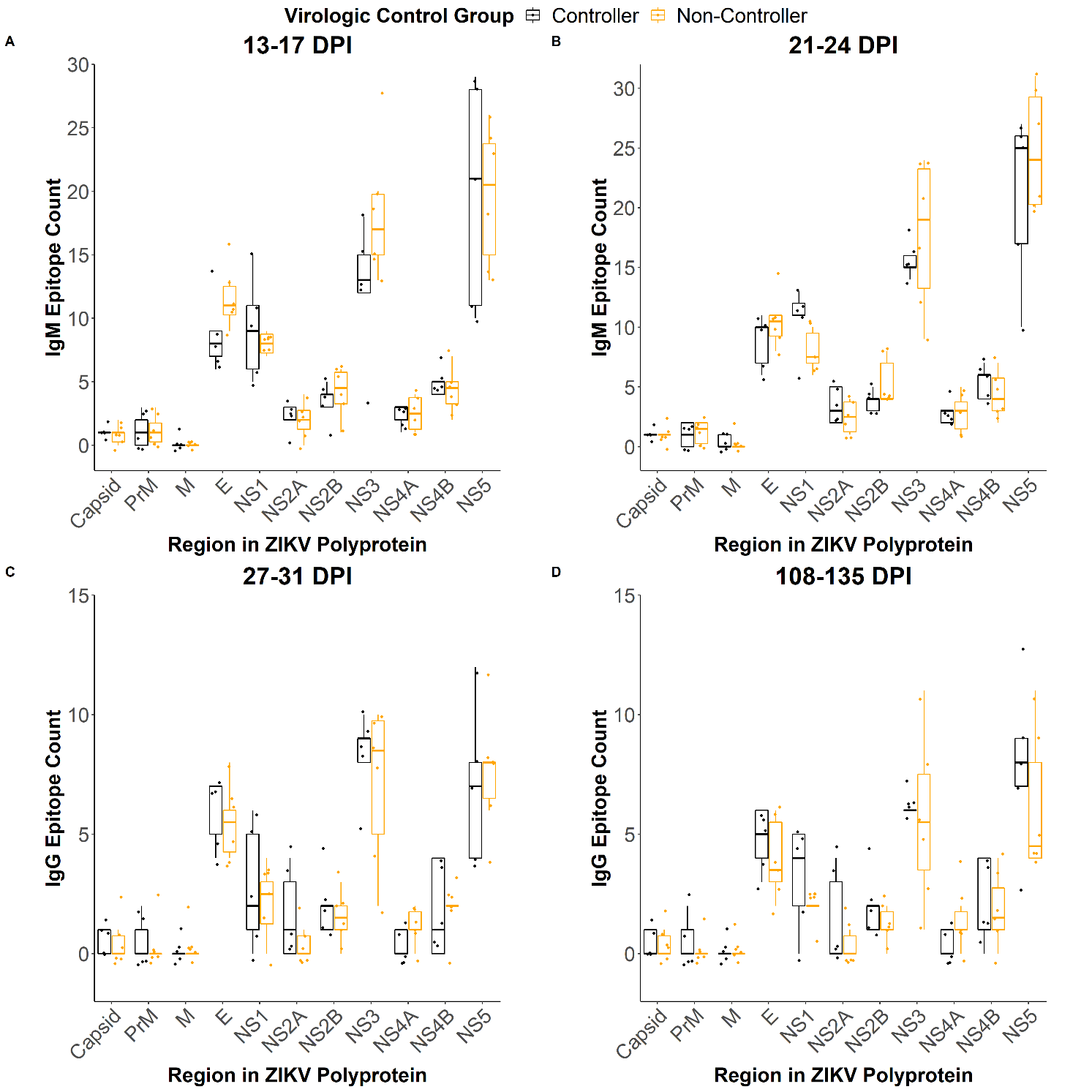


**Supplementary Figure 6. IgM and IgG linear epitope counts within each region of the viral polyprotein based on virologic control group.** Total IgM linear epitopes within each region of the ZIKV polyprotein were determined for each dam at **(A)** 13-17 days post-infection (DPI) and **(B)** 21-24 DPI. Similarly, total IgG linear epitopes within each region of the ZIKV polyprotein were determined for each dam at **(C)** 27-31 DPI and **(D)** 108-135 DPI. Dams are separated based on virologic control group. Statistical significance between virologic control groups was assessed using a Mann-Whitney U-Test (*p<0.05, **p<0.01, ***p<0.001).


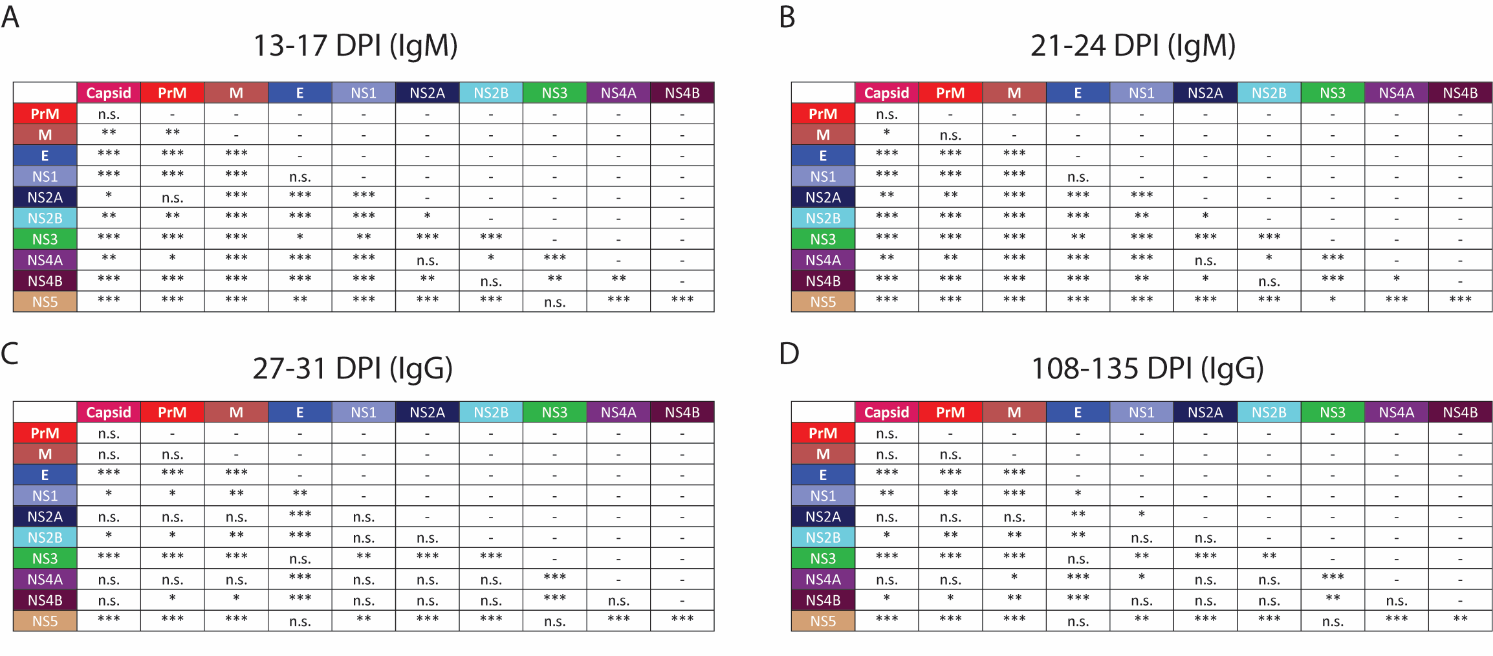


**Supplementary Figure 7.**  **Pairwise comparisons of linear IgM and IgG epitope counts within each region of the ZIKV polyprotein.** Total linear epitope counts were compared for each region of the ZIKV polyprotein at **(A)** 13-17 and **(B)** 21-24 days post-infection (DPI) for IgM and at **(C)** 27-31 and **(D)** 108-135 DPI for IgG. For this analysis, all dams were grouped into a single population and the linear epitope counts were determined. A Kruskal-Wallis test was used to look for significant differences across the regions of the ZIKV polyprotein at each time point followed by a pairwise Mann-Whitney U-test was used to identify specific proteins that significantly differ at each time point.  “n.s.” = not significant, *p< 0.05, **p< 0.01, ***p< 0.001.

**
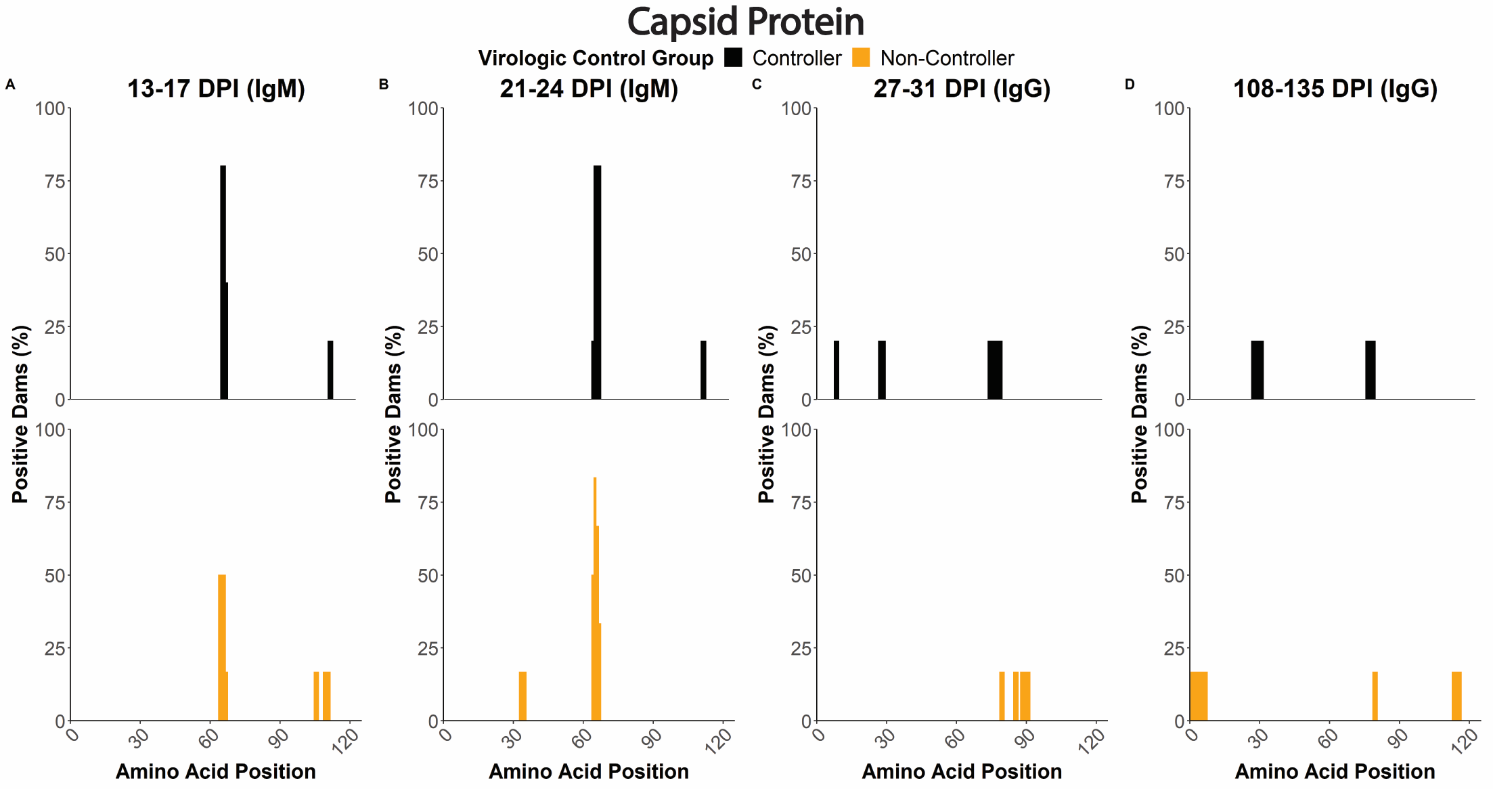
**

**Supplementary Figure 8. Capsid IgM and IgG linear epitope maps.** The percentage of dams exhibiting reactivity towards amino acids that make up the capsid linear epitopes was determined at **(A)** 13-17 and **(B)** 21-24 DPI for IgM and **(C)** 27-31 and **(D)** 108-135 DPI for IgG. The percentage of positive dams was determined based on virologic control group.

**
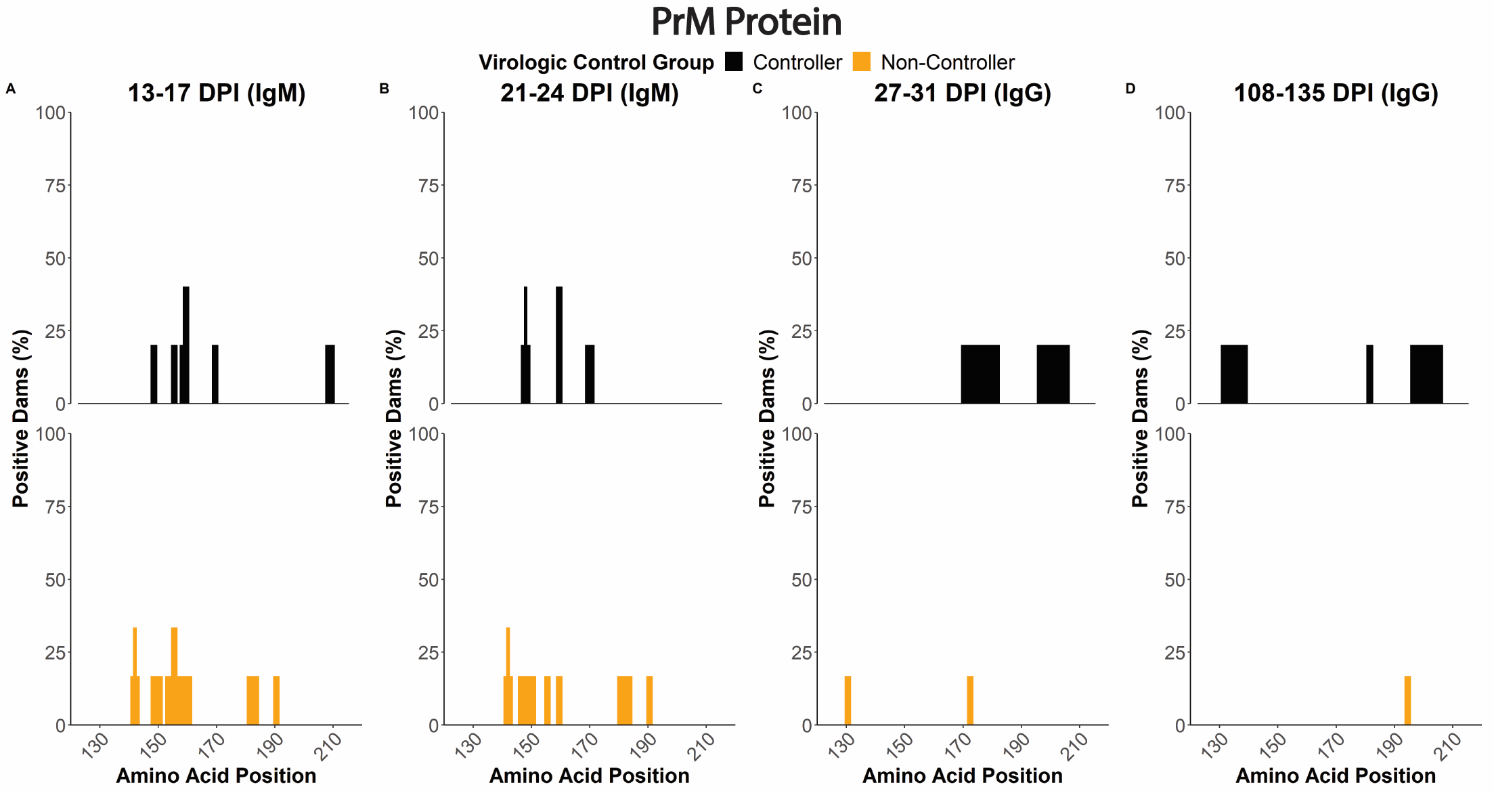
**

**Supplementary Figure 9. Pre-membrane (PrM) IgM and IgG linear epitope maps.** The percentage of dams exhibiting reactivity towards amino acids that make up the PrM linear epitopes was determined at **(A)** 13-17 and **(B)** 21-24 DPI for IgM and **(C)** 27-31 and **(D)** 108-135 DPI for IgG. The percentage of positive dams was determined based on virologic control group.

**
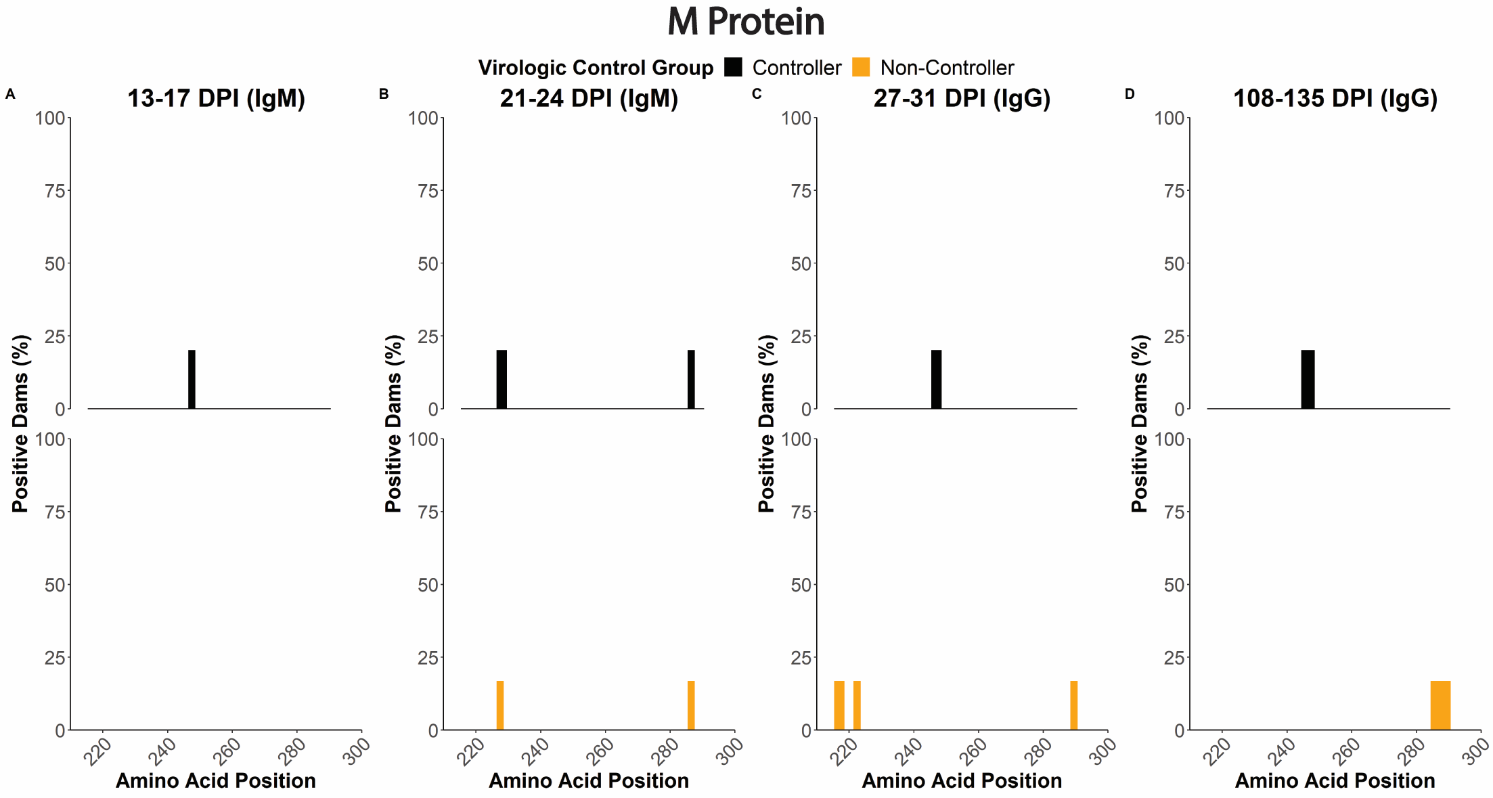
**

**Supplementary Figure 10. Membrane (M) IgM and IgG linear epitope maps.** The percentage of dams exhibiting reactivity towards amino acids that make up the membrane linear epitopes was determined at **(A)** 13-17 and **(B)** 21-24 DPI for IgM and **(C)** 27-31 and **(D)** 108-135 DPI for IgG. The percentage of positive dams was determined based on virologic control group.

**
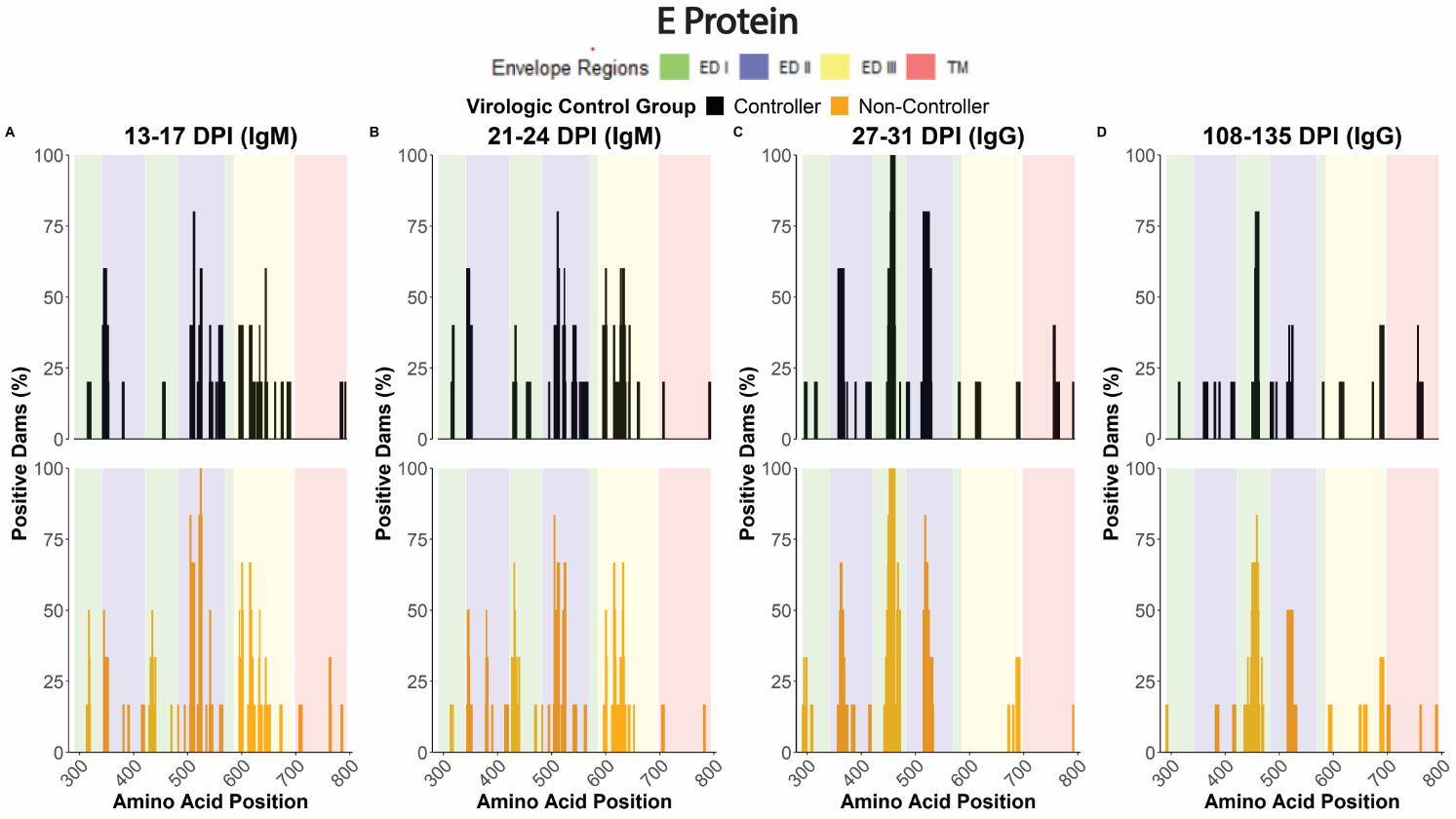
**

**Supplementary Figure 11. Envelope (E) IgM and IgG linear epitope maps.** The percentage of dams exhibiting reactivity towards amino acids that make up the envelope linear epitopes was determined at **(A)** 13-17 and **(B)** 21-24 DPI for IgM and **(C)** 27-31 and **(D)** 108-135 DPI for IgG. The percentage of positive dams was determined based on virologic control group. The individual sub-regions of the envelope protein are identified by different colors. “ED” = ectodomain, “TM" = transmembrane domain.

**
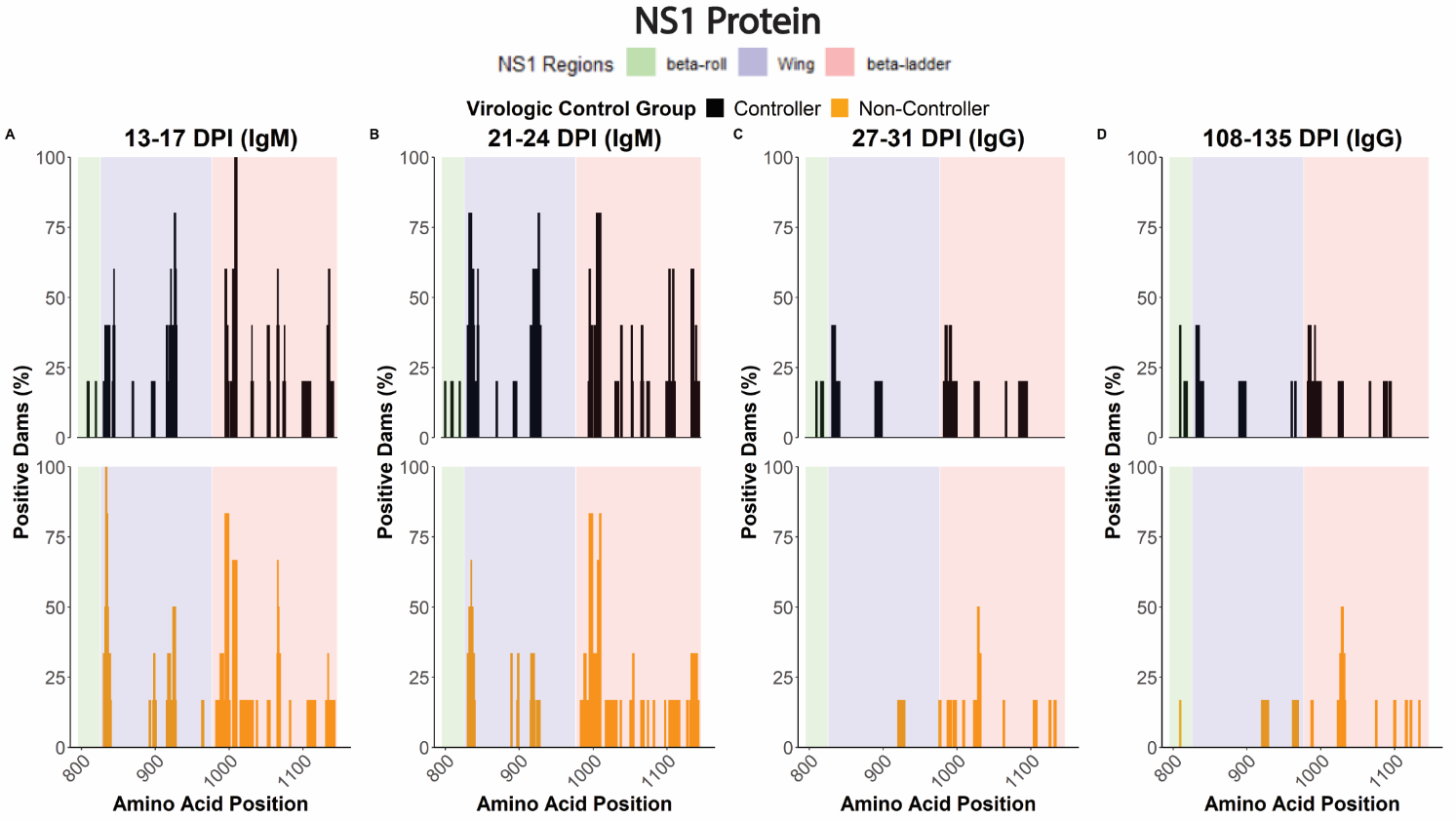
**

**Supplementary Figure 12. NS1 IgM and IgG linear epitope maps.** The percentage of dams exhibiting reactivity towards amino acids that make up the NS1 linear epitopes was determined at **(A)** 13-17 and **(B)** 21-24 for IgM and **(C)** 27-31 and **(D)** 108-135 DPI for IgG. The percentage of positive dams was determined based on virologic control group. The individual sub-regions of the NS1 protein are identified by different colors.

**
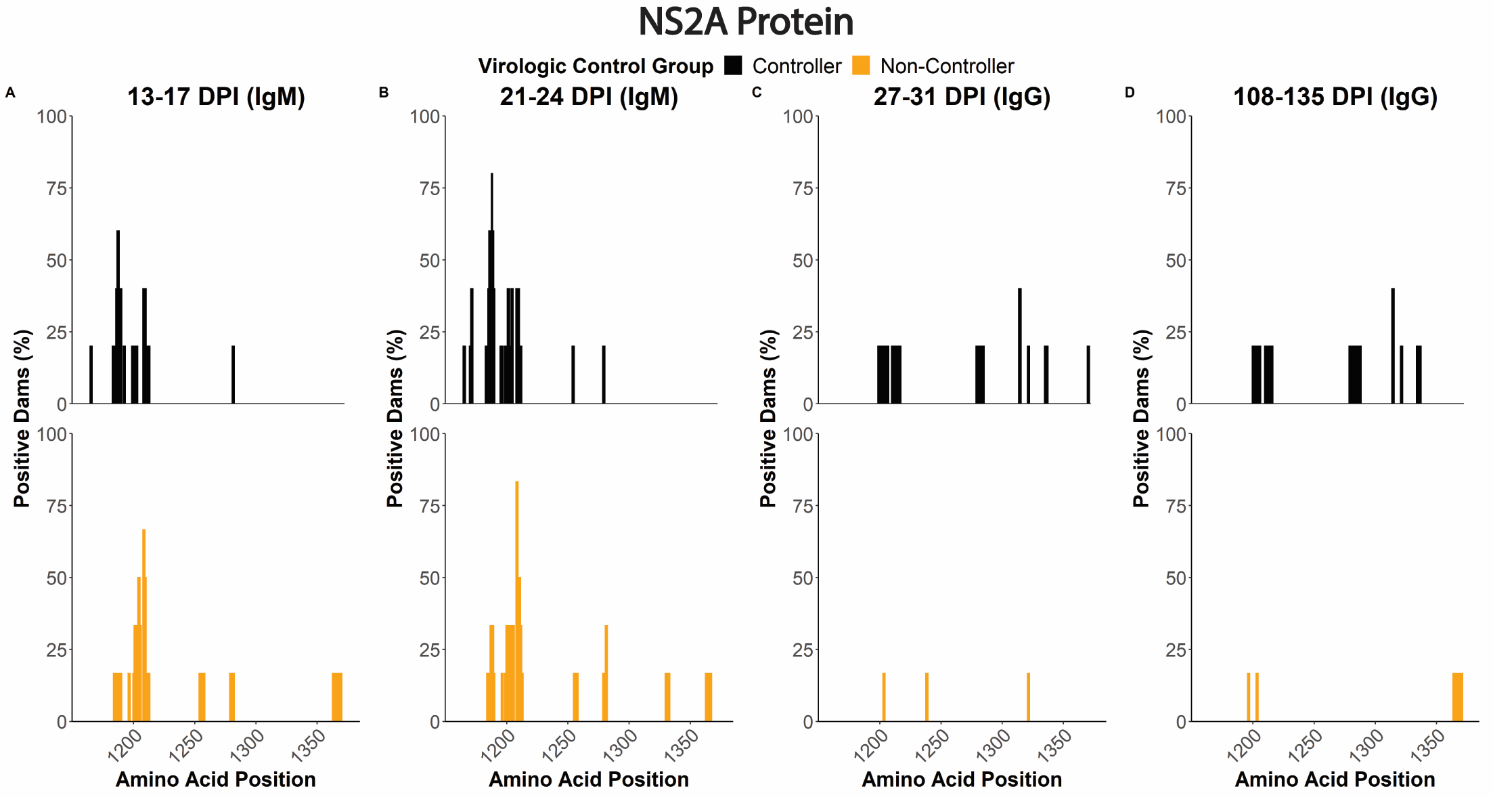
**

**Supplementary Figure 13. NS2A IgM and IgG linear epitope maps.** The percentage of dams exhibiting reactivity towards amino acids that make up the NS2A linear epitopes was determined at **(A)** 13-17 and **(B)** 21-24 DPI for IgM and **(C)** 27-31 and **(D)** 108-135 DPI for IgG. The percentage of positive dams was determined based on virologic control group.

**
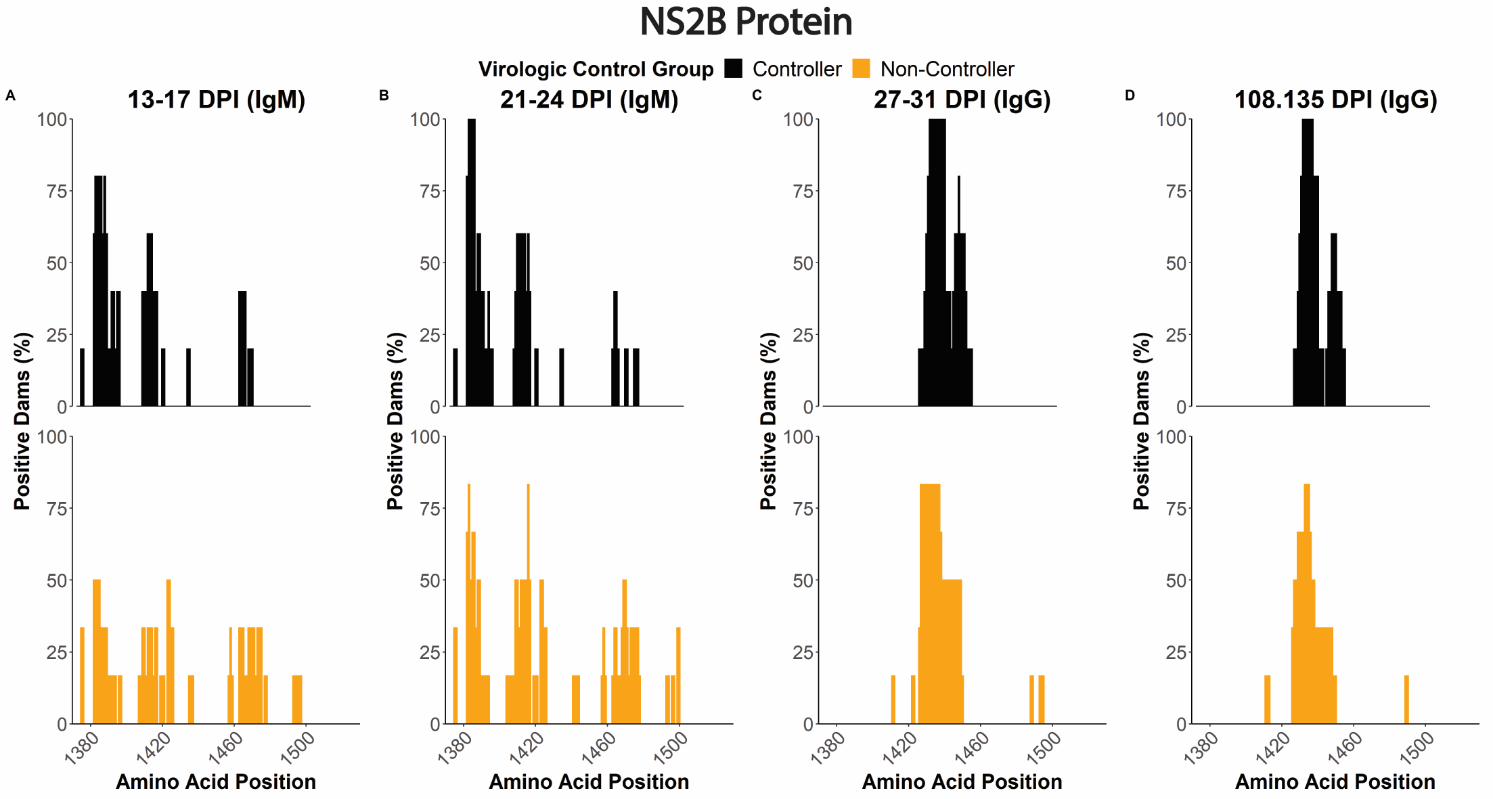
**

**Supplementary Figure 14. NS2B IgM and IgG linear epitope maps**. The percentage of dams exhibiting reactivity towards amino acids that make up the NS2B linear epitopes was determined at **(A)** 13-17 and **(B)** 21-24 DPI for IgM and **(C)** 27-31 and **(D)** 108-135 DPI for IgG. The percentage of positive dams was determined based on virologic control group.

**
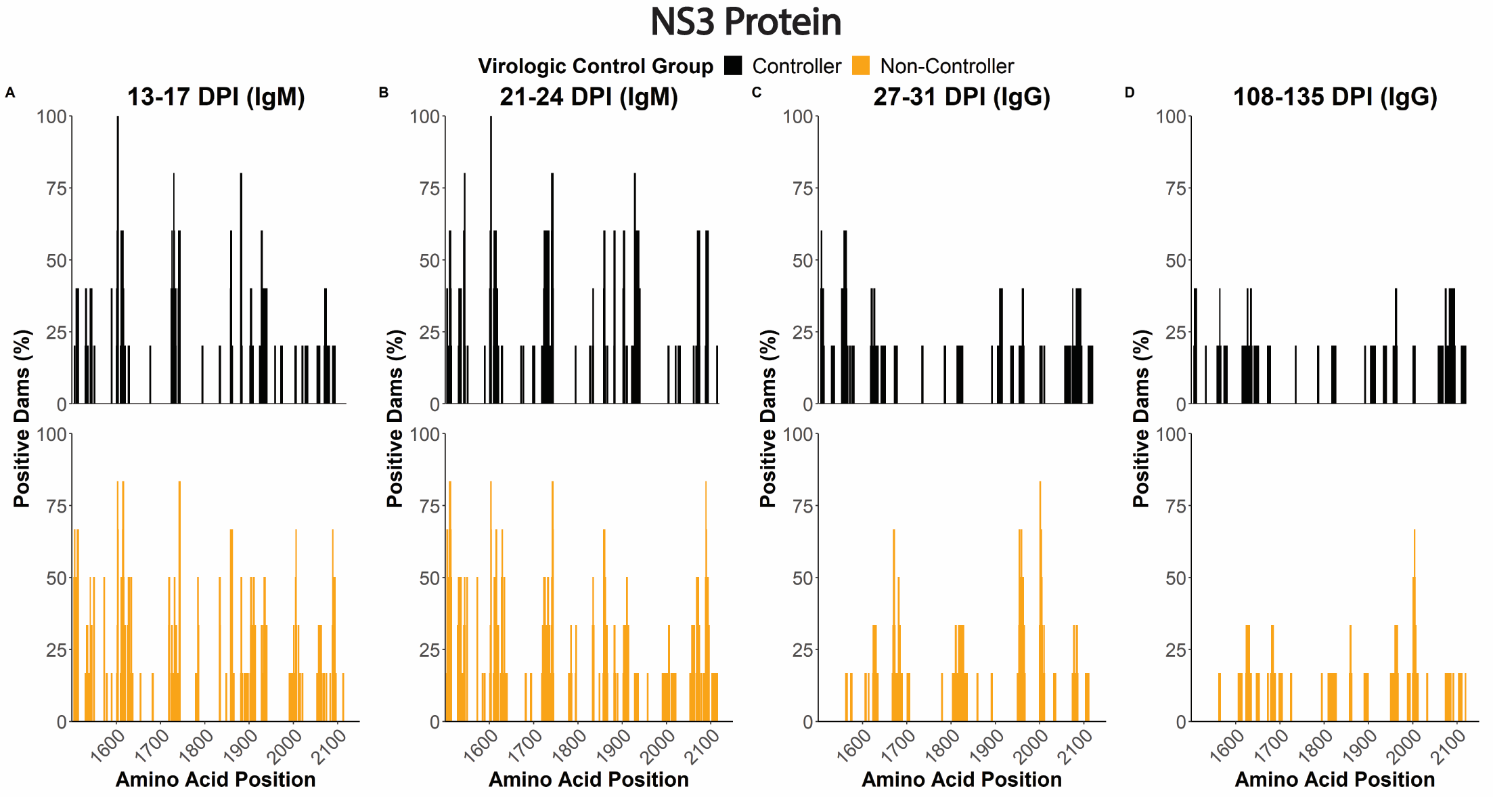
**

**Supplementary Figure 15. NS3 IgM and IgG linear epitope maps**. The percentage of dams exhibiting reactivity towards amino acids that make up the NS3 linear epitopes was determined at **(A)** 13-17 and **(B)** 21-24 DPI for IgM and **(C)** 27-31 and **(D)** 108-135 DPI for IgG. The percentage of positive dams was determined based on virologic control group.


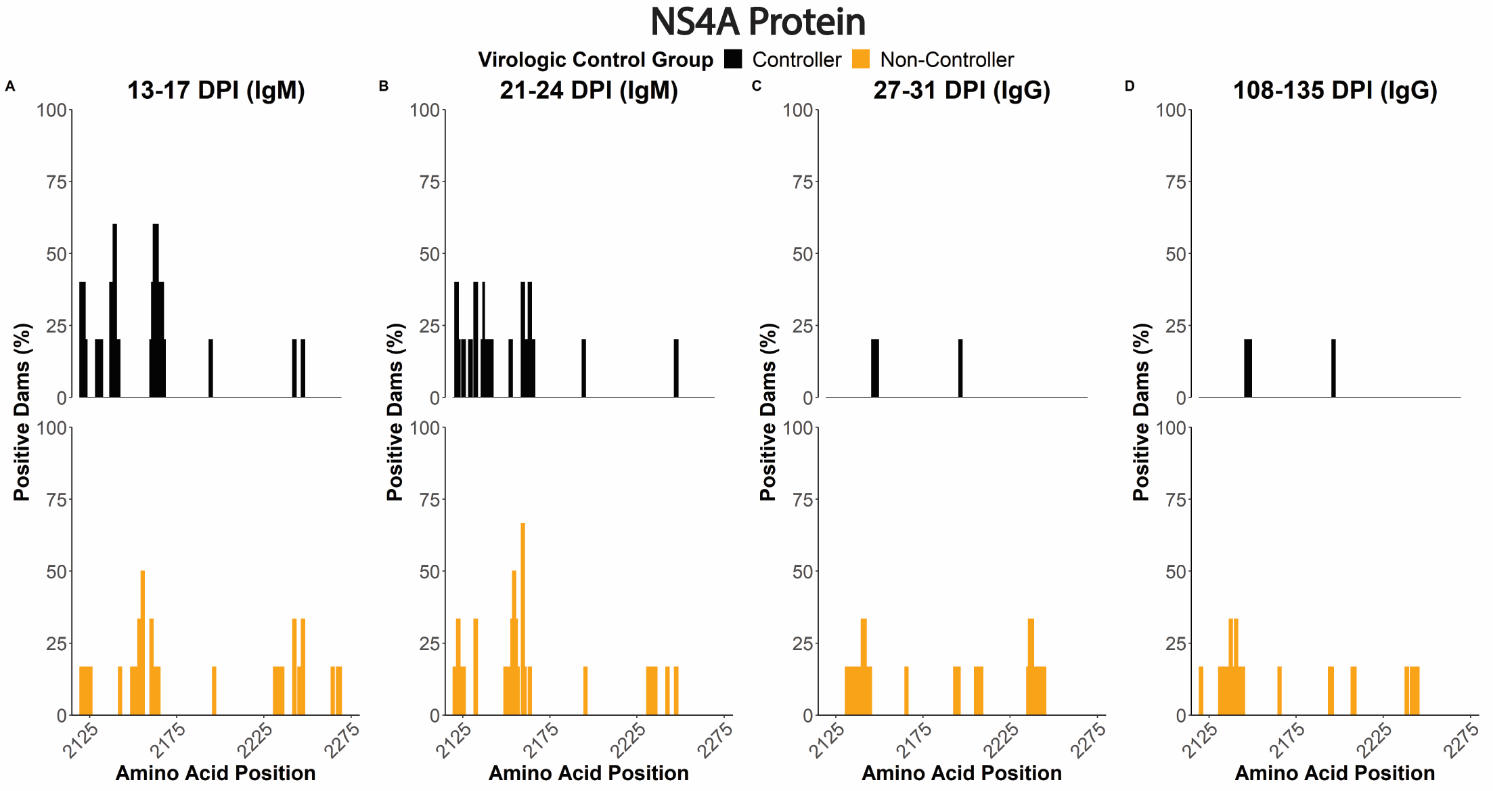


**Supplementary Figure 16. NS4A IgM and IgG linear epitope maps.** The percentage of dams exhibiting reactivity towards amino acids that make up the NS4A linear epitopes was determined at **(A)** 13-17 and **(B)** 21-24 DPI for IgM and **(C)** 27-31 and **(D)** 108-135 DPI for IgG. The percentage of positive dams was determined based on virologic control group.


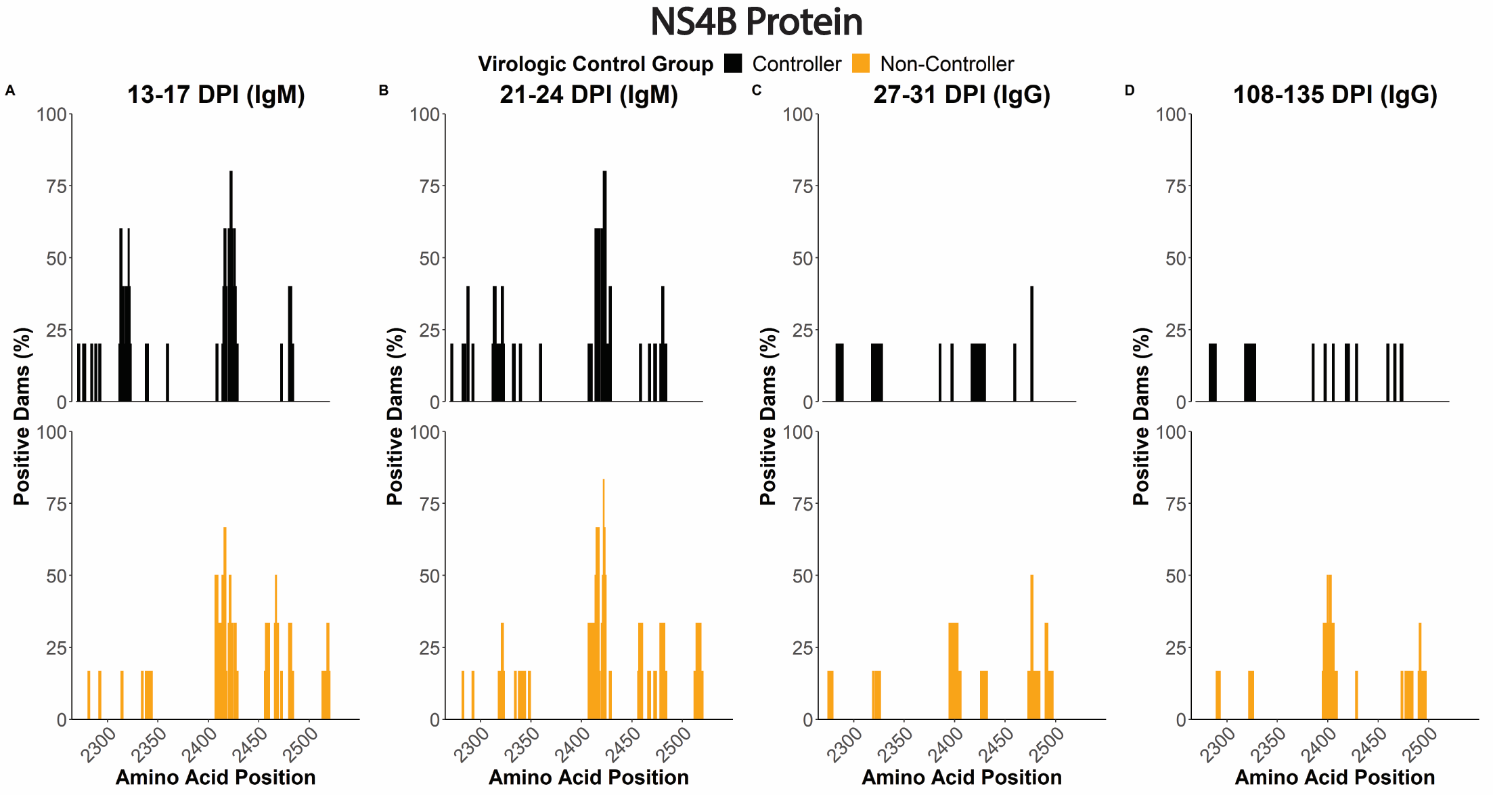


**Supplementary Figure 17. NS4B IgM and IgG linear epitope maps.** The percentage of dams exhibiting reactivity towards amino acids that make up the NS4B linear epitopes was determined at **(A)** 13-17 and **(B)** 21-24 DPI for IgM and **(C)** 27-31 and **(D)** 108-135 DPI for IgG. The percentage of positive dams was determined based on virologic control group.

**
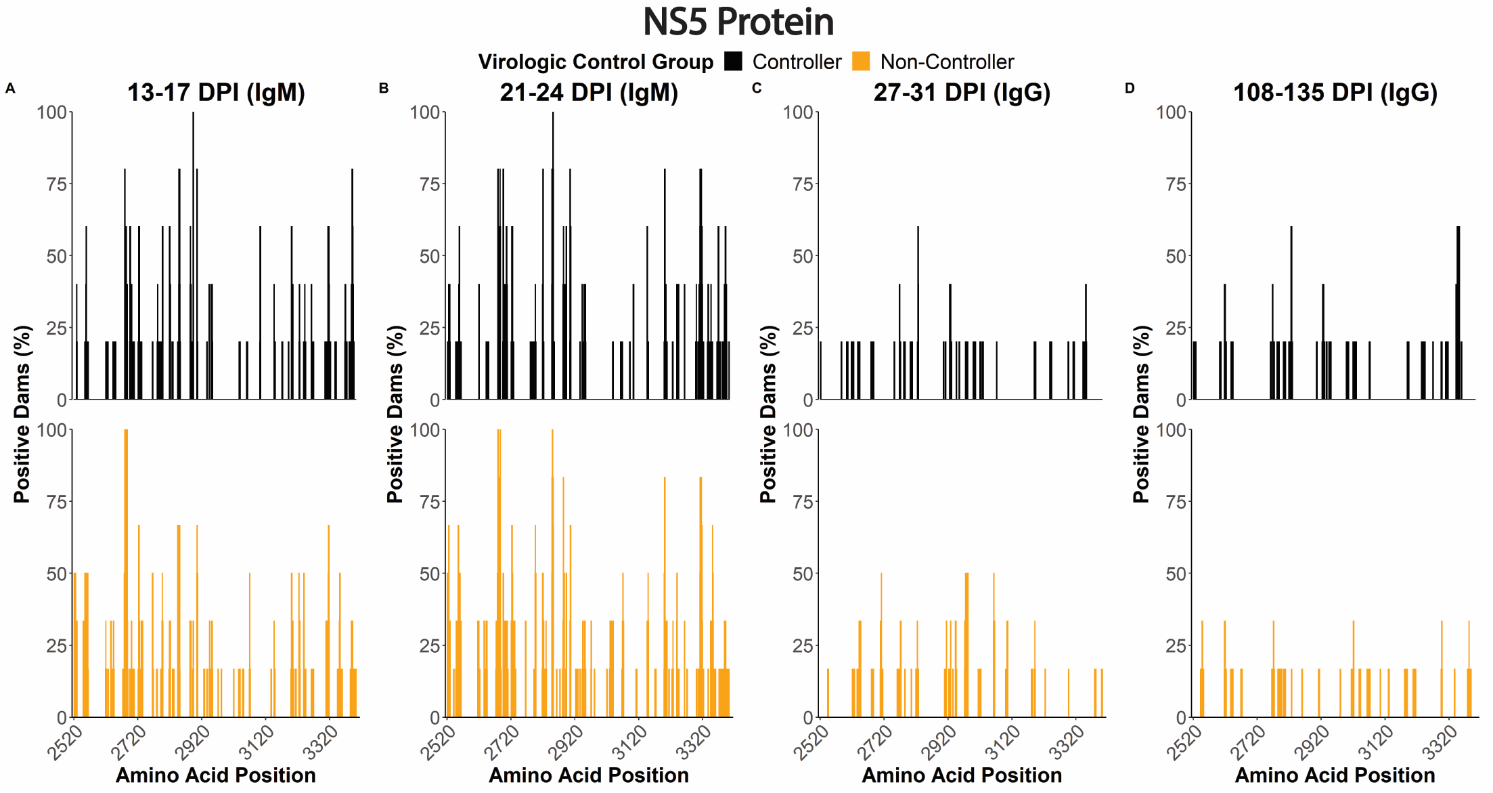
**

**Supplementary Figure 18. NS5 IgM and IgG linear epitope maps.** The percentage of dams exhibiting reactivity towards amino acids that make up the NS5 linear epitopes was determined at **(A)** 13-17 and **(B)** 21-24 DPI for IgM and **(C)** 27-31 and **(D)** 108-135 DPI for IgG. The percentage of positive dams was determined based on virologic control group.

## References

1. Pickett BE, Sadat EL, Zhang Y, Noronha JM, Squires RB, Hunt V, et al. ViPR: an open bioinformatics database and analysis resource for virology research. *Nucleic Acid Res* (2012) 40:D593-8. doi: 10.1093/nar/gkr859
